# Cross-country analysis of aggregate markups and their impact on income inequality.

**DOI:** 10.12688/f1000research.160674.4

**Published:** 2026-08-27

**Authors:** Peter Malliaros, W Alejandro Pacheco-Jaramillo

**Affiliations:** 1 Research, UrCommunity Ltd, Melbourne, VIC, 3051, Australia; 2 Economics, University Anahuac, Huixquilucan de Degollado, State of Mexico, 52786, Mexico

**Keywords:** Income Inequality (D63), Markups (D43), Economic Systems (P16), Research and Development (O32), Panel Data Analysis (C23), Tax Policy (H21)

## Abstract

**Background:**

This study investigates the connection between aggregate markups and income inequality, exploring how market power affects inequality in developed, emerging, and developing economies.

**Method:**

The study examines data from 12 countries from 1997 to 2016 using a panel data approach. A two-way fixed-effects specification is estimated after panel diagnostics for cross-sectional dependence, model selection, unit roots and cointegration. The analysis focuses on the Gini index, lagged markups, trade openness, GDP per capita, tax revenue and R&D expenditure.

**Results:**

Higher lagged markups are associated with greater income inequality, with the relationship particularly relevant for emerging and developing economies. Diagnostic checks support the use of fixed effects over random effects, while unit-root and cointegration tests indicate that the levels model should be interpreted as a long-run relationship rather than as a purely short-run association.

**Conclusion:**

The study shows how market power, fiscal redistribution and trade openness interact across diverse economic systems. The findings support competition policy and progressive taxation as complementary tools, while acknowledging that further dynamic panel and error-correction modelling would strengthen causal interpretation.

## Introduction

In recent years, the debate on the causes and consequences of rising income inequality has taken on considerable relevance in economic research. As the power of large companies has increased, with more concentrated markets in critical sectors, concern has arisen about how markups -or price margins that exceed production costs-influence income distribution and countries’ ability to reduce poverty effectively. High markups, which reflect the market dominance of large corporations, allow these companies to obtain greater profits, affecting market competitiveness and equity in the distribution of the wealth generated ( De Loecker & Eeckhout, 2018), understanding that significant gaps in economic inequality distort the market. Excessively high markups can also increase innovation, as by reducing market competition, companies have less incentive to invest in new developments and improve their products which I do not object, but I consider too much for the length of just one paper. This stagnation limits opportunities for inclusive growth and can reinforce income disparities. Our analysis focuses on how high markups affect income inequality, considering the role of various control variables. Understanding whether differences in markups across countries with different levels of development are associated with variations in income inequality is critical, as this relationship may reveal important insights into how market structures influence economic disparities and inform better public policies aimed at income redistribution.

This phenomenon becomes especially problematic in economies with limited competition in emerging and developing countries. However, the effects of markups are different; they depend on many aspects, such as each country’s institutional characteristics.

Economies with inclusive institutions that foster competition and regulate market power tend to have lower markups and more equitable income distribution ( Acemoglu & Robinson, 2023). In contrast, in countries with extractive institutions ^1^, High markups tend to concentrate wealth in the hands of a few companies, deepening inequality gaps and perpetuating poverty ( Stiglitz, 2020). Another example is that the lack of institutionalisation in developing countries generates high levels of corruption, subsequently leading to higher levels of inequality. It is like a vicious circle.

Although the impact of markups on macroeconomic dynamics has been widely studied, their direct relationship with income inequality across countries with different levels of development with different economic systems ^2^ ( Demir et al., 2022), has only recently begun to be explored in depth by several authors ( Lee & Lee, 2023; Han & Pyun, 2021; Bakir et al., 2021).

This analysis’s classification of tree groups of development and economic systems is crucial for understanding the institutional and policy frameworks that shape market dynamics and socio-economic outcomes in different contexts. Developed countries like the United States, Germany, Japan, and Australia operate under free-market systems supported by substantial property rights and competitive markets encouraging innovation. As North (1991) highlights, well-defined institutions such as the rule of law and property rights are fundamental to sustainable economic growth. Also, Robinson and Acemoglu (2012) emphasise that inclusive institutions ensure stability and help reduce extreme inequalities.

In contrast, emerging economies such as China, India, Mexico, and South Africa demonstrate hybrid systems that balance market-oriented reforms with strategic state interventions. Rodrik (2004) explains that these mixed approaches are common in economies transitioning to higher development levels, where the state plays a critical role in protecting key sectors and addressing market failures. One form of state intervention is through state-led initiatives, where the government actively promotes and supports industrial development, mainly where markets are underdeveloped. Similarly, Lin and Chang (2009) highlight the importance of such initiatives in fostering industrial development.

Developing countries, including Peru, Colombia, Venezuela, and the Philippines, often face the challenges of weak institutions and insufficient regulatory frameworks, which limit competition and exacerbate inequality ( Sen, 1999). However, as Stiglitz (2001) points out, this is not a permanent state. Markets tend to concentrate power in such environments, worsening income disparities, but with the right reforms, this can change. Easterly (2001) underscores how inconsistent policies and institutional limitations perpetuate poverty and inequality cycles and how these can be overcome. This classification underscores institutions’ varying roles in shaping economic systems and outcomes, offering hope for change.

This type of analysis is essential to understand how the structure of markets and institutional regulations affect the distribution of wealth and economic opportunities, especially among the most vulnerable households. Even though mainstream economists do not find a severe income inequality problem, it has many economic and social implications, including human behaviour.

When markups become excessively high, they can lead to inefficiencies, reducing consumer welfare and limiting economic mobility ( Hovenkamp, 2023; Enke, 1945). Recent research suggests that in developing countries, markups in essential sectors, such as food and energy, disproportionately affect low-income households, limiting their ability to access essential goods and thus exacerbating poverty levels ( Almunia & Antràs, 2019). Beyond the direct economic impact, high markups also have significant social consequences.

In contexts where income disparities are significant, people tend to experience a strong sense of injustice, which affects cooperation and trust in institutions and generates social and political instability ( Fehr et al., 1999).

This study aims to analyse the impact of aggregate markups on income inequality in a sample of developed, middle-income and developing countries. Data from 12 countries will be analysed, and the results will be distributed as follows: four developed countries, four middle-income countries, and four developing countries. They will be selected based on the economic system, institutional diversity, development level, and information availability. The countries considered in this analysis include the United States, Germany, Japan, India, Mexico, the Philippines, Colombia, South Africa, and Australia, representing a broad range of continents and cultures, with data covering the period from 1997 to 2016. This time interval will allow observing these two decades’ long-term effects and structural changes.

The methodological approach will be a panel data econometric analysis, which will capture variations over time and across countries. To separate the specific effects of markups on inequality, factors such as GDP per capita growth, research and development, R&D as a percentage of GDP, trade openness, taxes on income, trade openness and poverty will control the model. Poverty levels are reflected in the percentage of the population living below the international poverty line. Robustness tests will also be carried out to ensure the robustness of the results, along with a comparative analysis between economies to identify possible differences in the effects of markups in both contexts.

## Theoretical framework

Income inequality is associated with various economic and social challenges, such as reduced economic mobility, social instability, and obstacles to long-term growth ( Stiglitz, 2012; OECD, 2015). It restricts access to essential resources for lower-income groups and exacerbates economic divides, resulting in less inclusive economic outcomes ( Atkinson, 2015; Piketty, 2014). The link between markups and income inequality has been the subject of intense debate in the current economic literature, with several recent studies providing clear empirical evidence of this relationship across different countries and sectors ( Han & Pyun, 2021; Lee & Lee, 2023; Bakir et al., 2021). Markups reflect the ability of firms to set prices above their production costs, which indicates their market power. In highly competitive markets, this ability is limited, but in sectors where few firms predominate, or there is a high concentration, markups tend to be much higher ( De Loecker & Eeckhout, 2018). This increase in the power of large firms has led many economists to reflect on their impact on income distribution and inequality.

High markups contribute to inefficiency by inflating consumer prices, which reduces purchasing power and worsens income inequality. They indicate lower competition, leading to misallocated resources and stifled innovation, as firms experience less pressure to improve. Recent studies have investigated the complex interplay between innovation, market power (markups), and income inequality, highlighting that innovation can amplify market power when dominated by a few leading firms, consequently worsening income inequality ( Aghion et al., 2021; Eeckhout et al., 2024). This specific relationship will be tested empirically using R&D expenditure as a control variable, allowing us to capture its indirect impact on income distribution.

Aghion et al. (2019) analysed U.S. data, and they found that increased innovation correlates with a higher income share for the top 1%, indicating that innovation may exacerbate income inequality at the upper end of the distribution. Karagiannis and Tsoulfidis (2019) extended this analysis globally, concluding that while innovation incentivises economic growth, it often leads to greater income inequality, mainly when its benefits are unevenly distributed.

Guellec and Paunov (2017) focused on digital innovation, arguing that technological advancements can result in market concentration and increased market power for leading firms, thereby heightening income inequality. Similarly, Aghion et al. (2021) found that increased market concentration, often driven by innovation, can stifle competition and contribute to rising income inequality. These studies suggest that although innovation drives economic progress, it can increase income inequality, mainly when market power is concentrated among a few dominant firms. However, addressing the dominance of large technological oligopolies requires a balanced approach that combines fostering innovation with implementing antitrust policies. By encouraging new firms to enter the market through reduced regulatory hurdles and strategic support, the competitive landscape can shift, challenging the market power of established players ( Spulber, 2023). At the same time, antitrust measures are crucial to curbing monopolistic practices, such as unfair pricing strategies or restrictive competition policies. This dual approach helps drive prices closer to production costs, benefiting consumers and smaller businesses. Furthermore, innovation-focused initiatives can enhance sector-wide productivity, offering diverse and competitive alternatives to the entrenched influence of tech giants. Together, these measures ensure a more equitable and dynamic market environment while safeguarding against excessive concentration of power.

Additionally, high markups create barriers to market entry, which diminishes economic dynamism and hampers equitable wealth distribution ( Autor et al., 2020; De Loecker & Eeckhout, 2018; Syverson, 2019). According to De Loecker & Eeckhout (2018), the upward trend in markups over the last two decades reflects how market power has concentrated in a small group of large corporations, especially in technology, telecommunications and pharmaceuticals. By controlling the market, these companies can set higher prices, generating higher profit margins. These profits tend to be concentrated in the hands of the owners of capital, reducing the proportion of income received by workers and consumers. According to the last authors, these gaps contribute to increased income inequality. Subsequently, why is it not so suitable to have significant gaps in economic inequality?

Thomas Piketty (2014) analyses how this dynamic has powered inequality over time. In his work on capital in the 21st century, Piketty argues that the return on capital has consistently exceeded the rate of economic growth, which has allowed those who already own capital (shareholders and business owners) to see their wealth grow at a faster pace than the rest of the population. In this context, markups play a central role by allowing large firms to capture a larger share of the wealth generated, thereby intensifying the disparities between those who own capital and those who depend on their wages. Also, it is even worse in Latin America, which has a greater concentration of wealth in a few, making it the most unequal continent.

On the other hand, Milton Friedman and neoliberal economists have long argued that this pricing power is temporary ( Friedman, 1962). In their view, competitive markets should regulate themselves, as the entry of new firms would reduce markups over time. However, Joseph Stiglitz (2001) criticises this view, arguing that, in practice, market concentration and weak competition policies have allowed markups to remain high, thus contributing to rising inequality. Stiglitz emphasises that market failures that enable markups to persist will not be automatically corrected and that the resulting inequalities require more active intervention. Understanding this analysis, the effects of income inequality are very different in the three distinct groups of countries, and the impact is less in developed countries due to their strong economies and better institutions.

Grouping countries in an economic system provides valuable insight into the interplay between markups and income inequality. A country’s economic framework (whether focused on free-market principles, state-driven policies, or a mixed approach) directly impacts competition, market entry, and income redistribution ( Tybout, 2000). Developed nations, with their robust institutions, often mitigate the adverse effects of high markups more effectively. In contrast, emerging and developing economies frequently grapple with persistent high markups due to weaker institutions. Incorporating this variable allows a deeper understanding of how different economic systems shape the connection between markups, inequality, and overall economic performance.

We hypothesise that countries with lower levels of income inequality tend to have smaller markups than those with higher inequality. In such economies, prices often align more closely with production costs, promoting a competitive market and making goods and services more accessible for all income groups ( Moon et al., 2011). This balanced pricing structure may help prevent wealth from concentrating at the top, as firms possess less power to set high markups, and wages are likely to be more evenly distributed. Consequently, higher income inequality may be associated with economies where markups are more significant, primarily benefiting a few dominant firms.

Monopolies and oligopolies are like the big players in the game where competition barely exists, letting one or just a few companies take the lead. In a monopoly, a single company holds all the cards, setting prices as high as they want because there is no one else in the ring to challenge them ( Stigler, 1988). Oligopolies are not much different; with just a handful of firms calling the shots, prices can soar, and consumer choices dwindle ( Tirole, 1988). This lack of competition means these companies have little motivation to innovate or cut costs, leading to a stale market where consumers do not get the best products or prices ( Schmalensee, 1989). Also, these dominant players often create obstacles that keep newcomers out, ensuring their reign remains unchallenged and leaving consumers with fewer options ( Carlton & Perloff, 2005).

The outcome from these market dynamics stretches far beyond just higher prices—it significantly contributes to growing income inequality. When companies earn significant profits through high markups, that wealth often lands in the hands of a select few executives and shareholders. At the same time, everyday workers and consumers bear the impact ( Galbraith, 1998). This concentration of wealth creates a barrier for many, making it challenging for them to move up the economic ladder while those at the top keep reinforcing their status ( Autor et al., 2020). In addition, with less competition, workers have diminished bargaining power, leading to stagnant wages and fewer benefits. This cycle of inequality continues to widen the gap between the rich and the rest of society ( Atkinson, 2015).

The role of public institutions in moderating market power cannot separate the debate on markups from Acemoglu & Robinson (2023), this reference is Robinson and Acemoglu (2012) who have proposed that inclusive institutions tend to promote competition and, therefore, limit firms’ ability to set high prices on a sustained basis. According to these authors, when institutions are solid and transparent, the market is regulated more equitably, reducing the concentration of power and improving income distribution. For instance, good practices in competitive markets can be found in the firms of Nordic European countries.

This approach contrasts with Mancur Olson’s view (1982), which argues that modern societies are frequently captured by interest groups that manage to influence public policies to protect their economic interests. In such contexts, extractive institutions are created, which favour elites and perpetuate the concentration of market power. From this perspective, high markups not only reflect a lack of competition but also the ability of these elites to influence the rules of the game in their favour. Thus, robust institutions in developed countries play a crucial role in curbing such influence, ensuring fair competition, and fostering a more balanced economic environment.

Stiglitz (2020) reinforces this thinking by highlighting that market power is tolerated and often encouraged in many countries with extractive institutions. In these cases, governments and the most potent companies cooperate to perpetuate income concentration, preventing a fairer wealth distribution. For Stiglitz, public institutions play a crucial role in moderating the market: when these institutions are transparent and oriented toward the common good, they promote competition and, in doing so, limit the excessive accumulation of market power. Considering that the classical economy is based on incentives, the capitalist does not have the role of looking after social interests, nor should they; state institutions must look after social policies such as a better distribution of resources, whether through taxes, subsidies or social policies. For instance, taxes are crucial in improving income distribution and reducing societal inequality. Taxes are a primary mechanism through which governments can redistribute wealth, funding essential public services like education, healthcare, and social welfare programs that benefit lower-income individuals and families ( Barr, N. 2020). Progressive tax systems, where higher earners pay more of their income, can effectively reduce disparities by reallocating resources to those in greater need ( Rybakov et al., 2022). Moreover, the relationship between direct taxes and GDP can provide valuable insights ( Maganya, 2020). Our study investigated how effectively these 12 countries manage their income distribution. Including this relationship in our model could reveal how direct taxation changes correlate with income inequality shifts, enhancing our understanding of fiscal policy’s impact on economic equity ( Pacheco-Jaramillo, 2023; Gunasinghe et al., 2021).

Douglass North (1990) offers another perspective, arguing that institutions affect competition and the market and foster trust. North notes that trustworthy and transparent institutions facilitate more efficient and fair economic transactions. On the other hand, in markets where markups remain high due to a lack of competition, distrust in institutions can lead to financial and social instability.

The impact of markups in developing countries is even more acute, as lower-income consumers are especially vulnerable to high prices in essential sectors. In Latin America, if we consider an average salary of 400 USD per month for an operator compared to a manager’s 20,000 USD, the income gap exceeds 5,000%. While the manager’s role may justify higher pay, this disparity can seem unfair to lower-wage workers, affecting their motivation ( Statista, 2023). In the 12 countries analysed, high markups contribute to wealth concentration, especially in settings with limited competition, poor institutional quality, and low investment in innovation. Richard Thaler (2017) argues that people care not only about their income but also about how they compare to others, so when inequality is visible, it generates frustration that can lead to reduced cooperation. Daniel Kahneman (2002) reinforces this by showing that social comparison directly impacts perceived well-being, where visible inequality can lead to dissatisfaction and political instability. Similarly, Ernst Fehr and Schmidt (1999) suggests that individuals may sacrifice their income to “punish” perceived injustices, indicating that high markups and concentrated wealth can erode social cohesion and economic stability across these nations. Almunia and Antràs (2019) show that high markups on food and energy impose a disproportionate burden on low-income households, who spend a more significant share of their resources on these goods in emerging markets. The lack of competition in these sectors allows large companies to dominate the market and maintain high prices, perpetuating poverty in these economies ( Sachs, 2005).

Similarly, Monga and Ndulu (2020) analyse how high markups in strategic sectors, such as energy and telecommunications, restrict the access of the poorest households to essential services in sub-Saharan Africa. These authors highlight that the lack of access to affordable services perpetuates poverty and limits people’s ability to participate in the economy more productively, trapping them in cycles of economic exclusion.

Although some economists, such as Paul Collier (2007), have argued that the solution could lie in increasing trade openness and foreign direct investment, other authors, such as  Ha-Joon Chang (2008), warn that without adequate regulation, trade openness could lead to new forms of market concentration. Chang suggests that trade liberalisation without safeguards could allow large foreign companies to capture the local market, potentially exacerbating inequality and poverty problems. There is no dispute that there are many opportunities when there is open trade or for the entry of foreign investment. The problem is that these openings could generate more inequality in countries with poor institutionality.

Our theoretical framework retains its broad scope to contextualize the relationship between market power, innovation, and income inequality across institutional contexts. However, we explicitly acknowledge that certain theoretical elements, while relevant, are not directly incorporated into our empirical analysis. This intentional choice ensures clarity in the empirical model and maintains methodological rigor, providing justification for excluding specific factors, such as behavioural aspects, that do not align closely with the empirical structure presented.

## Methods

This study investigates the relationship between aggregate markups with income inequality and control variables across 12 countries for 20 years, from 1997 to 2016. By integrating data from multiple global and national sources, we aim to provide a comprehensive and nuanced understanding of how market power influences inequality across diverse economic and institutional contexts. Our approach combines well-established international databases. This combination of international databases ensures we capture various economic realities, from advanced economies with robust markets to developing countries with more concentrated sectors and weaker institutions. The data for the variables used in this analysis is sourced directly from the World Bank database platform ( https://databank.worldbank.org), where users can filter information by variable and period as needed. However, the data related to markups or aggregate markups is exclusively obtained from the National Bureau of Economic Research database NBR, specifically from the work of De Loecker, J., and J. Eeckhout (2018): “The Rise of Market Power and the Macroeconomic Implications,” published as an NBER working paper. This dataset is available on their official website ( https://sites.google.com/site/deloeckerjan/data-and-code) under the title “Aggregate markups from Global Market Power: Country-year (xls) and Continent-year (xls).” This source provides detailed country-level and continent-level data on aggregate markups, which is critical for macroeconomic analyses of global market power.

Our analysis focuses on income inequality as the key dependent variable, measured through the Gini coefficient. We include markup and control variables (  Table 1) to account for other factors influencing inequality, such as GDP per capita, poverty, trade openness, innovation percentage of GDP and taxes on income (direct taxes). GDP per capita purchasing power parity (PPP) is used because it offers a consistent way to compare economic well-being across countries by adjusting for price differences and inflation. This measure ensures that variations in purchasing power and living standards are accurately reflected and free from currency distortions.

**Table 1.** Variables.

Variable - Acronyms	Description	Source
Income Inequality - INE (Dependent Variable)	Measures income distribution disparities in each country using the Gini index.	World Bank ( CEIC Data, 2024)
Aggregate Markups - MARK	This is the ratio of price to marginal cost in product markets. It is the difference between the cost of a good or service and its selling price, expressed as a percentage above the cost.	National Bureau of Economic Research (NBER)
Taxes on income, profits and capital gains (% of revenue)	Constitute the portion of total tax revenue, which is crucial for understanding how tax policies contribute to the economy.	World Bank
GDP per capita, PPP (constant 2021 international $)	This indicator reflects the average economic output per person, adjusted for purchasing power and inflation, enabling fair comparisons across countries. It provides a standardised measure of living standards and economic well-being.	World Bank
Trade Openness – TO	Indicates the level of economic integration and the country's openness to global trade, impacting inequality and markups.	World Bank
Poverty – POV	The poverty headcount ratio at $2.15 is the percentage of the population living on less than $2.15 daily at 2017 international prices. It reflects the percentage of the population living below the poverty line, indicating economic hardship levels.	World Bank
Innovation as % of GDP – RD	It represents the investment in research and development relative to the country's total economy. It is a widely used indicator to assess a country's commitment to innovation.	World Bank
Economy System Category	1. **Market economies** primarily with strong regulatory frameworks and well-established institutions (US, Germany, Japan, Australia) 2. **Mixed economies**, predominantly blending market mechanisms with significant state intervention in strategic sectors (China, India, Mexico, South Africa) 3. **State Capitalism, ** largely state-influenced or state-led economies, characterised by weaker institutions, less mature markets, and heavier government control (Philippines, Venezuela, Colombia and Peru)	International Monetary Fund’s ( IMF, 2025), *World Economic Outlook (WEO)*

Three groups were established to classify countries: developed countries, emerging countries (  Table 2), and developing countries—this classification aimed to identify associations or patterns between income inequality, markups, and various controlled variables. Their economies’ stability and the Gini coefficient’s volatility were also considered. For instance, the Gini coefficient in developing countries often exhibits significant fluctuations linked to government changes. The table below presents the motivation behind selecting the 12 countries analysed.

**Table 2.** Characteristics and types of economic models in twelve countries.

Country	Description	Economic Development
United States	As the world's largest economy, the U.S. provides a key benchmark for understanding how market power drives inequality and poverty, especially with markups growing in the tech and pharma sectors.	Advanced Economy (OECD)
Germany	Europe’s industrial powerhouse is highly competitive yet still shows markups in manufacturing and energy. In contrast to other economies, its regulatory frameworks are solid.	Advanced Economy (OECD)
Japan	Unique industrial strength and regulated market provide insights into markups in slow-growing sectors, making it ideal for exploring institutional interactions.	Advanced Economy (OECD)
Australia	As an advanced economy with significant resource exports, Australia's markups in sectors like mining and energy provide insights into how concentrated markets affect inequality in a country with solid institutions.	Advanced Economy (OECD)
South Africa	As Africa's most industrialised nation, South Africa faces extreme income inequality, reflected in its high Gini coefficient. Its concentrated markets in mining and energy make it ideal for studying the relationship between markups and income distribution.	Emerging Economy (BRICS)
Mexico	High trade openness combined with concentrated energy and telecom sectors where markups persistently drive inequality. Geographical ties with the U.S. offer added context.	Emerging Economy (OECD)
China	China’s rapid growth, significant state involvement, and market concentration in crucial industries like technology and energy make it a critical case for understanding markups and inequality in a fast-developing economy.	Emerging Economy (BRICS)
India	Largest developing economy where markups in agriculture and energy hit poor populations hardest. A multi-faceted case for studying markups and poverty in diverse settings.	Emerging Economy (BRICS)
Peru	Latin America's rapidly developing economy relies heavily on mining and natural resources. With persistent income inequality and market concentration in key sectors, it offers valuable insights into how markups affect economic disparity.	Developing Economy (LatAm)
Philippines	Facing challenges such as income inequality, infrastructure gaps, and reliance on sectors like agriculture and remittances, which are typical characteristics of developing economies.	Developing Economy (Asia)
Venezuela	With one of the world's largest oil reserves, it has a highly centralised economy heavily reliant on petroleum. Its severe income inequality and market distortions provide a unique case for studying the relationship between markups and economic disparity.	Developing Economy (LatAM)
Colombia	Colombia, a diverse and growing economy in Latin America, is marked by high-income inequality and significant market concentration in sectors like energy and telecommunications.	Developing Economy (LatAM)

By the authors.

After selecting the variables and the countries, we test the association between the markup and income inequality and its controlled variables through an econometric panel model. Thus, we will examine variables like aggregate markups, taxes on income, GDP per capita PPP, trade openness, poverty and innovation.

### Descriptive analysis

**Figure 1.**
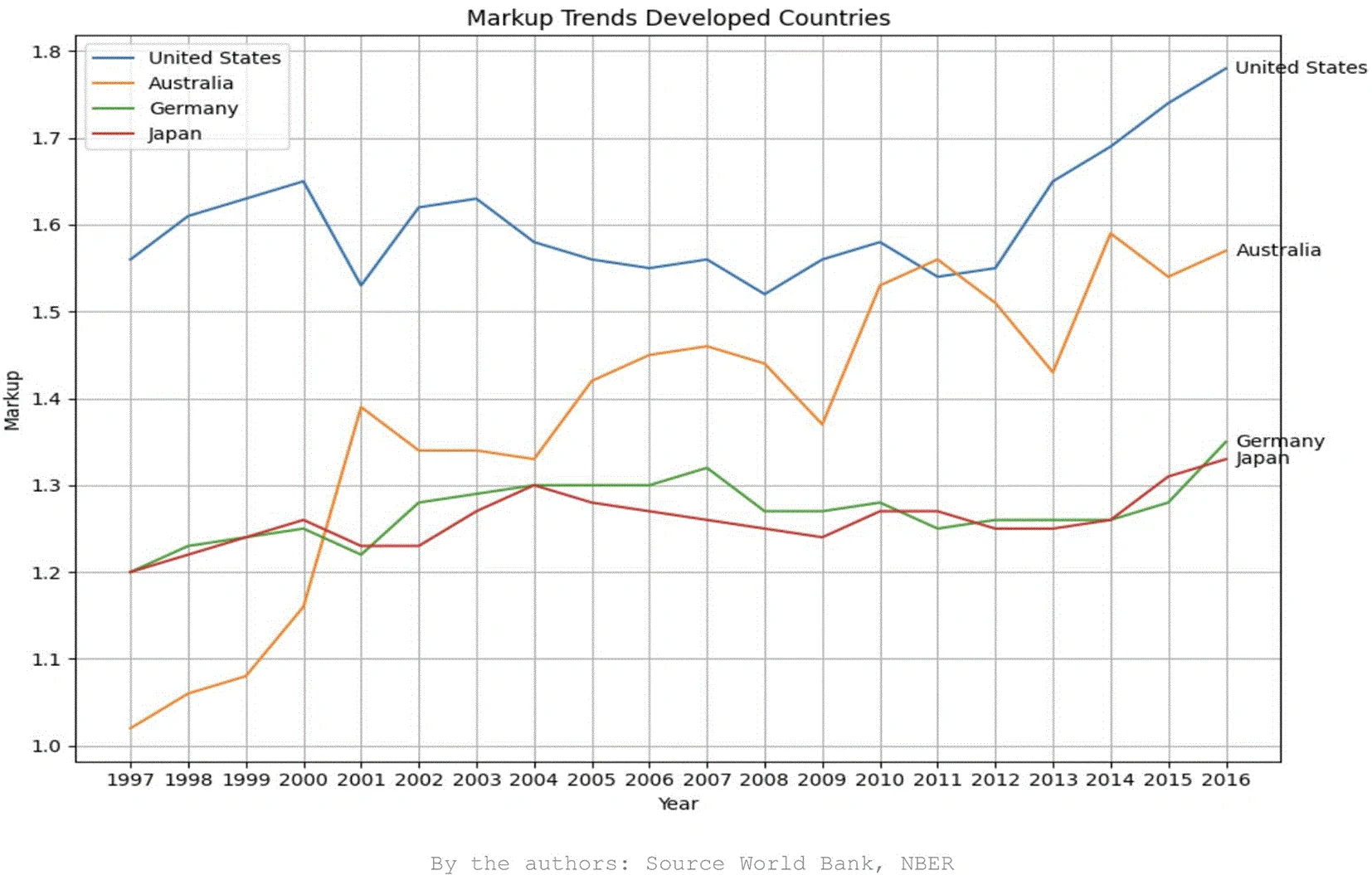
Markup trends developed countries. Source: National Bureau of Economic Research (NBER). By the authors.

The markup trends across the 12 analysed countries reveal distinct dynamics, highlighting differences between developed, emerging, and developing economies. In developed economies, markup increases tend to be gradual and stable as shown in  Figure 1. For instance, the United States saw its markup rise from 1.56 in 1997 to 1.78 by 2016, while Germany’s markup showed a steadier trend, increasing from 1.20 to 1.35 over the same period. Australia, however, stands out with a sharper upward trend, where its markup grew significantly from 1.02 in 1997 to 1.57 in 2016, suggesting increased market power within specific sectors. These changes reflect relatively well-regulated markets where additional revenue capture does not lead to extreme income disparities.

**Figure 2.**
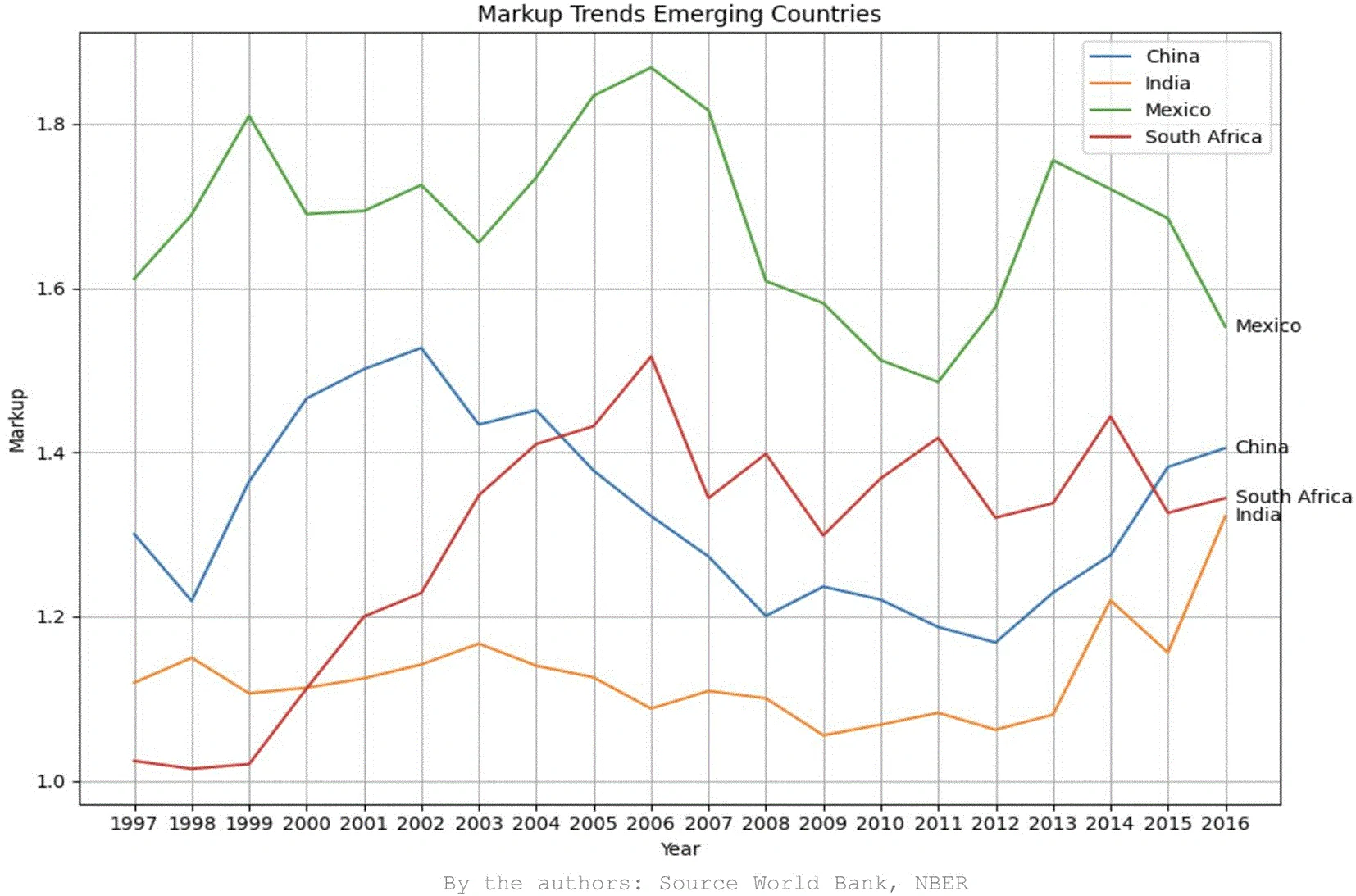
Markup trends emerging countries. Source: National Bureau of Economic Research (NBER). By the authors.

Emerging economies like China, India, Mexico, and South Africa display more varied trends as shown in  Figure 2. China experienced a markup decline from 1.55 in 2002 to 1.19 in 2012, indicating improving market competition in average. India, in contrast, saw fluctuations before its markup rose to 1.32 by the end of the period. Mexico’s markup remained higher than many other countries, peaking at 1.86 in 2010 and stabilising around 1.55 by 2016. South Africa exhibited an upward trajectory, with its markup increasing from 1.02 in 1997 to 1.34 in 2016, reflecting market power consolidation in critical industries. These trends suggest that while some emerging markets are moving toward greater competition, others continue to face challenges from concentrated market power.

**Figure 3.**
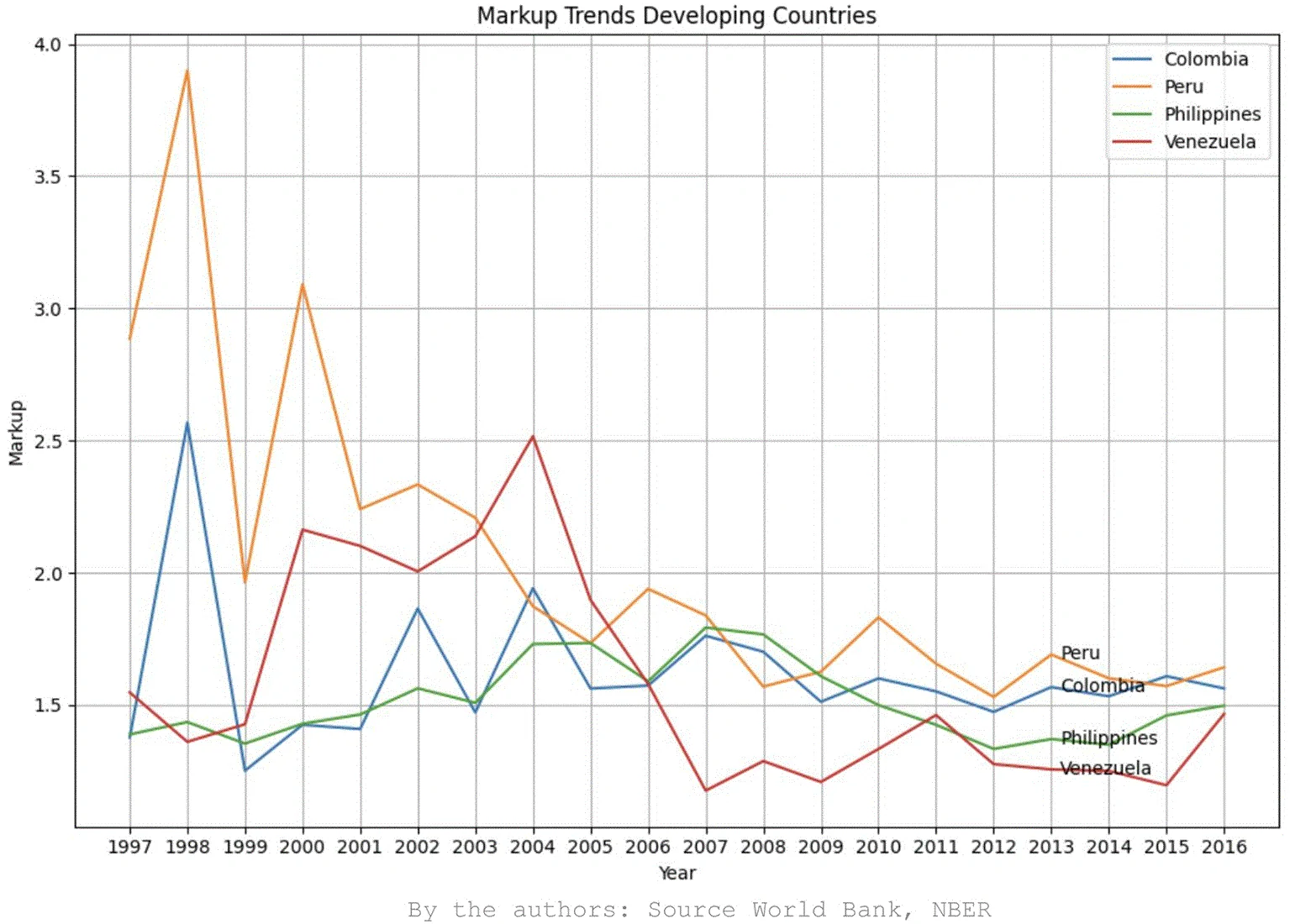
Markup trends developing countries. Source: National Bureau of Economic Research (NBER). By the authors.

Developing economies show even greater volatility, often experiencing dramatic markup swings as shown in  Figure 3. Peru, for example, peaked at 3.9 in the early 2000s before declining to 1.6 by 2016, indicating significant market restructuring. Similarly, Venezuela exhibited swings from 2.5 to 1.2, reflecting economic instability and weaker regulatory frameworks. In contrast, the Philippines maintained relatively stable markups, fluctuating between 1.3 and 1.7. Colombia saw a marked decline, with its markup decreasing from 2.5 to approximately 1.6, pointing to improved regulatory environments. These patterns suggest that in developing countries, reducing high markups could align with policies aimed at income redistribution. However, the econometric analysis will assess how these markups interact with income inequality and control variables.

**Figure 4.**
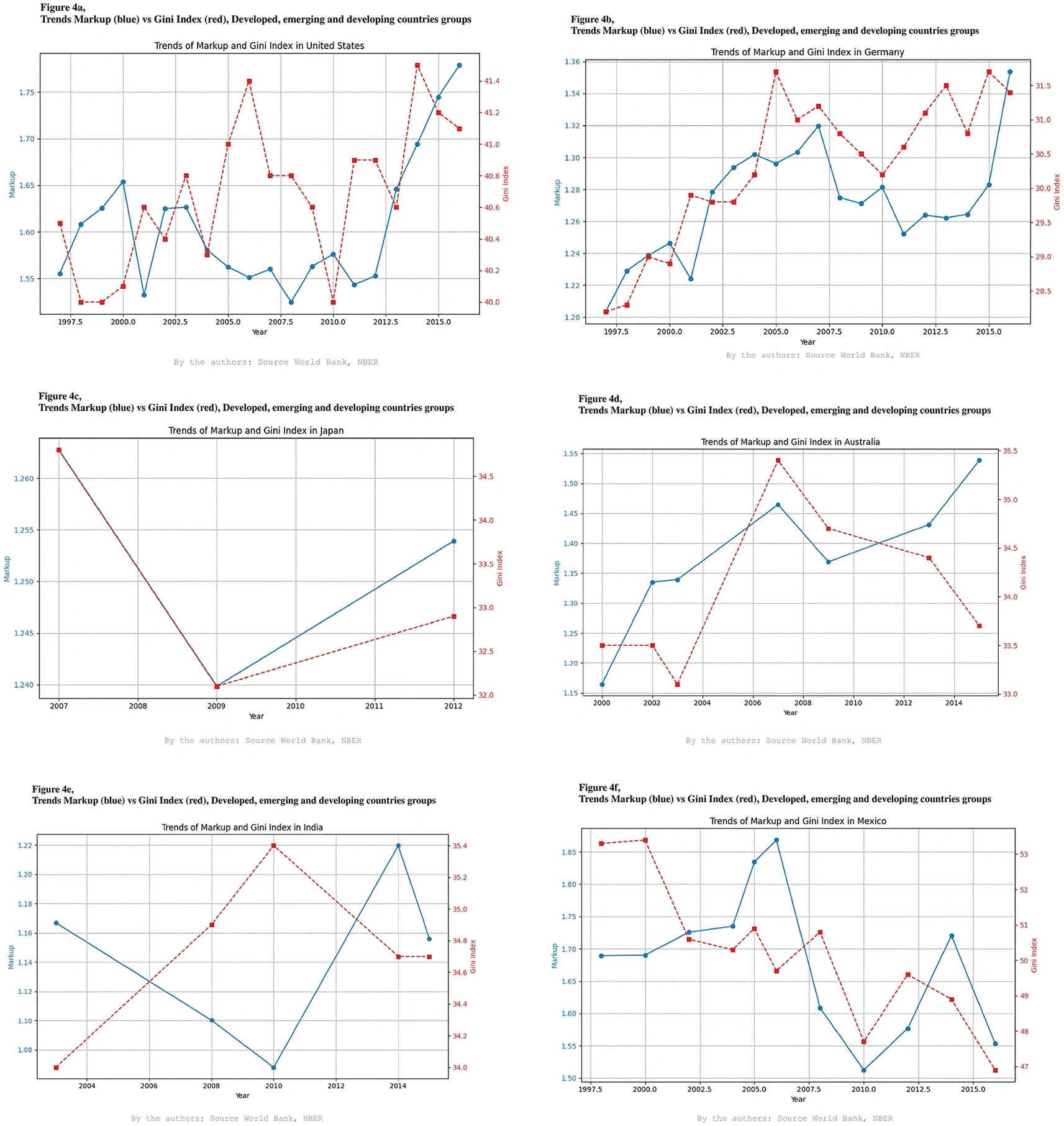

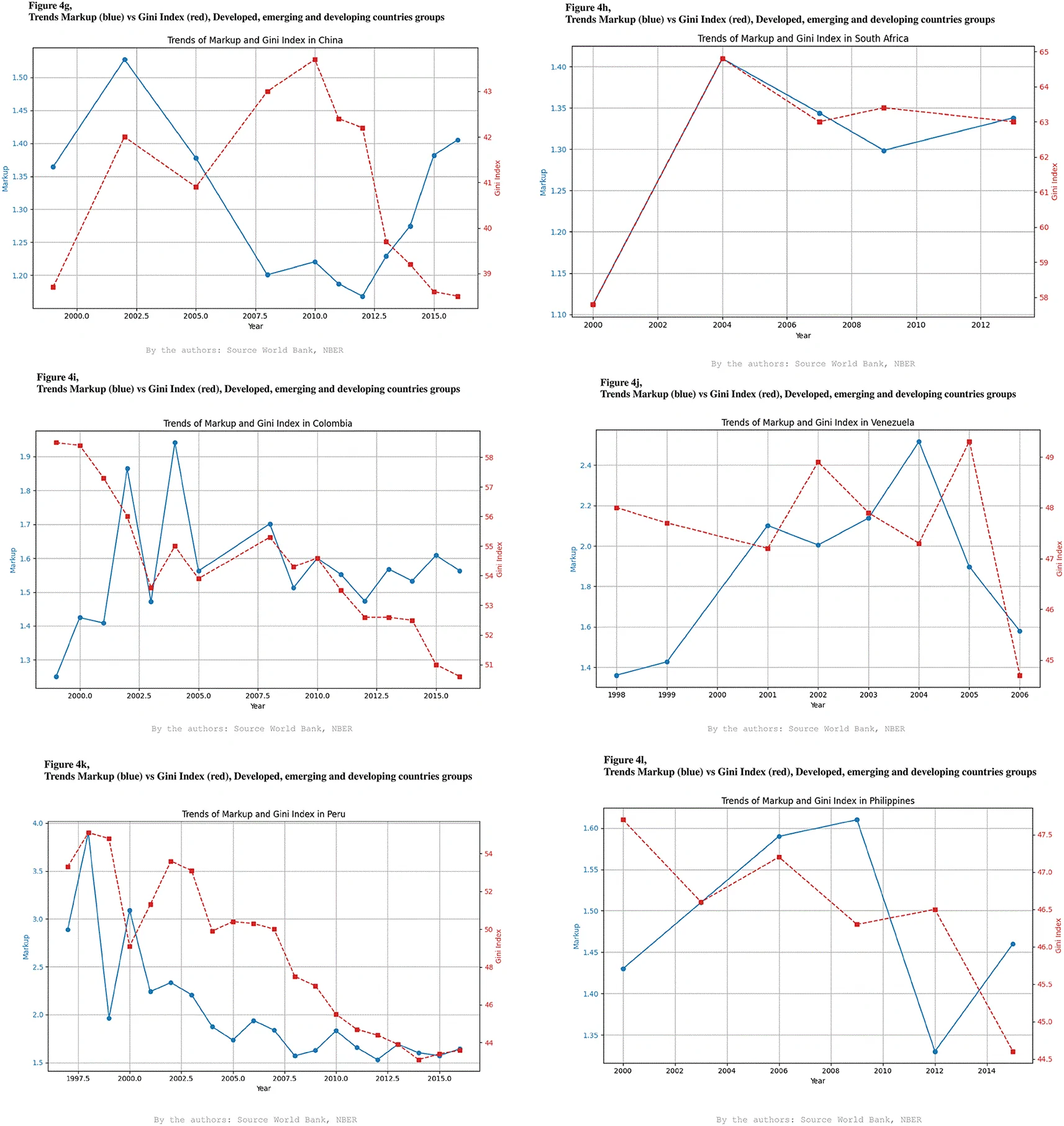
Trends Markup (blue) vs Gini Index (red), Developed, emerging and developing countries groups.

### Markup vs Gini index

While the relatively stable increase in markups in developed economies does not significantly exacerbate income inequality, it does not act as a mechanism to reduce it. Despite higher markups reflecting increased market power and profitability in specific sectors, the additional revenues are not necessarily channelled towards reducing income disparities. Instead, they often benefit shareholders and executives, leaving the underlying income distribution unchanged. The stability in inequality observed in developed countries is more likely the result of robust redistributive policies and social safety nets than the impact of markup dynamics.

In developed countries like the United States, Germany, and Australia, we noticed a trend: income inequality increased as companies made more profit. For example, in Australia, the profit margin grew significantly from 1.01 in 1996 to 1.56 in 2016, about a 54% increase. The gap between the rich and poor widened slightly during the same period. This suggests that when businesses are doing well and earning higher profits, the extra wealth might not be shared evenly among everyone, leading to greater income inequality.

Moving on to emerging economies such as China, India, Mexico, and South Africa, the picture is less clear. In Mexico, company profit margins increased until around 2010, peaking at 1.86, and then declined. Interestingly, income inequality has shown a declining trend since 1998, suggesting other factors like fiscal and social policies might have overshadowed the direct effect of markups on inequality ( Colciago & Mechelli, 2020).

This could mean that other factors—like government policies, economic reforms, or social programs—play a significant role in how income is distributed, overshadowing the impact of company profits.

Company profit margins and income inequality have decreased in developing countries like Peru, Colombia, Venezuela, and the Philippines. Take Peru, for example: from 1997 to 2016, the profit margin dropped from 2.89 to 1.64, and income inequality decreased significantly. This might indicate that as companies have lower profit margins, wealth is being spread more evenly among the population, reducing the gap between the rich and the poor.

**Figure 5.**
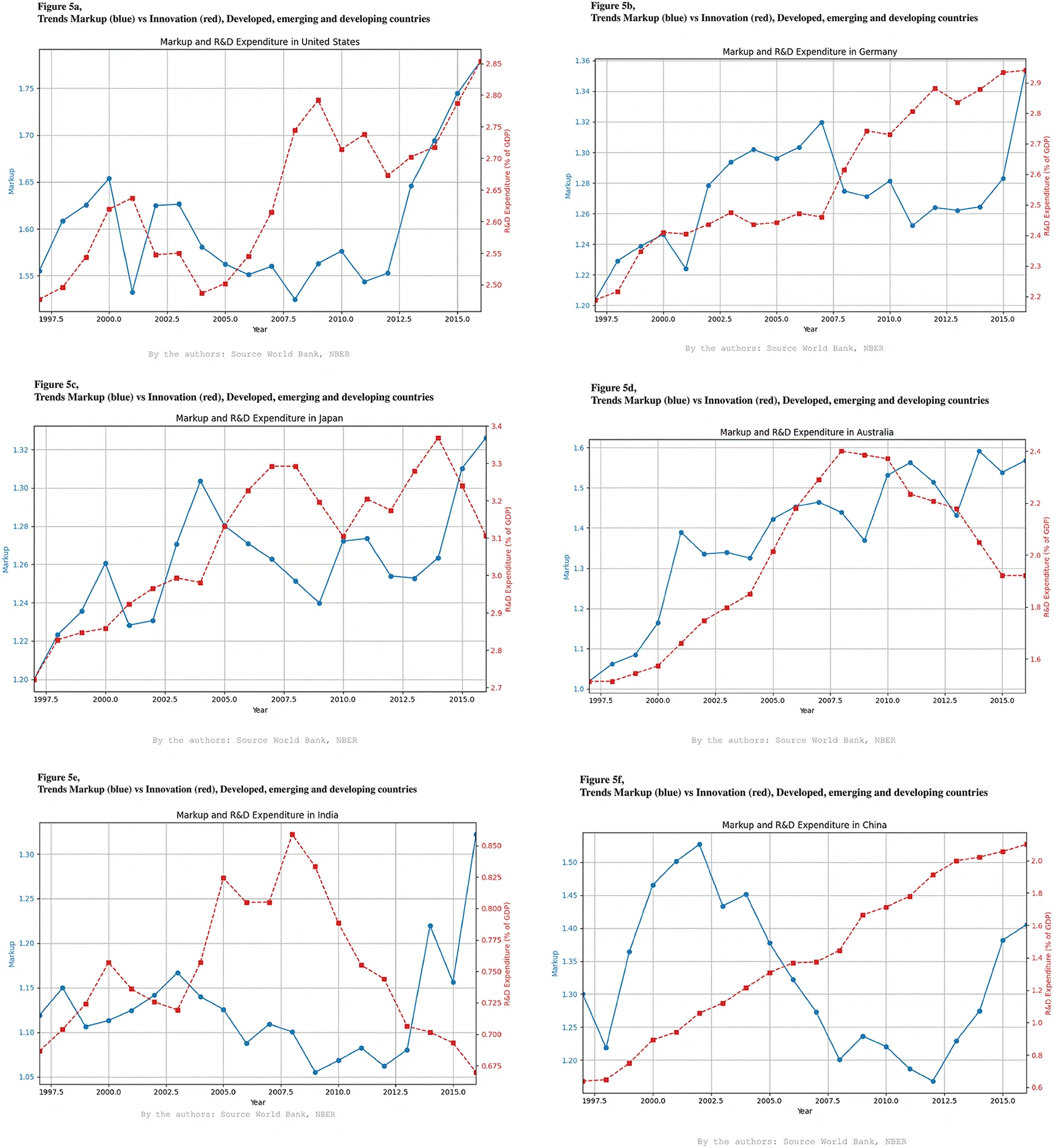

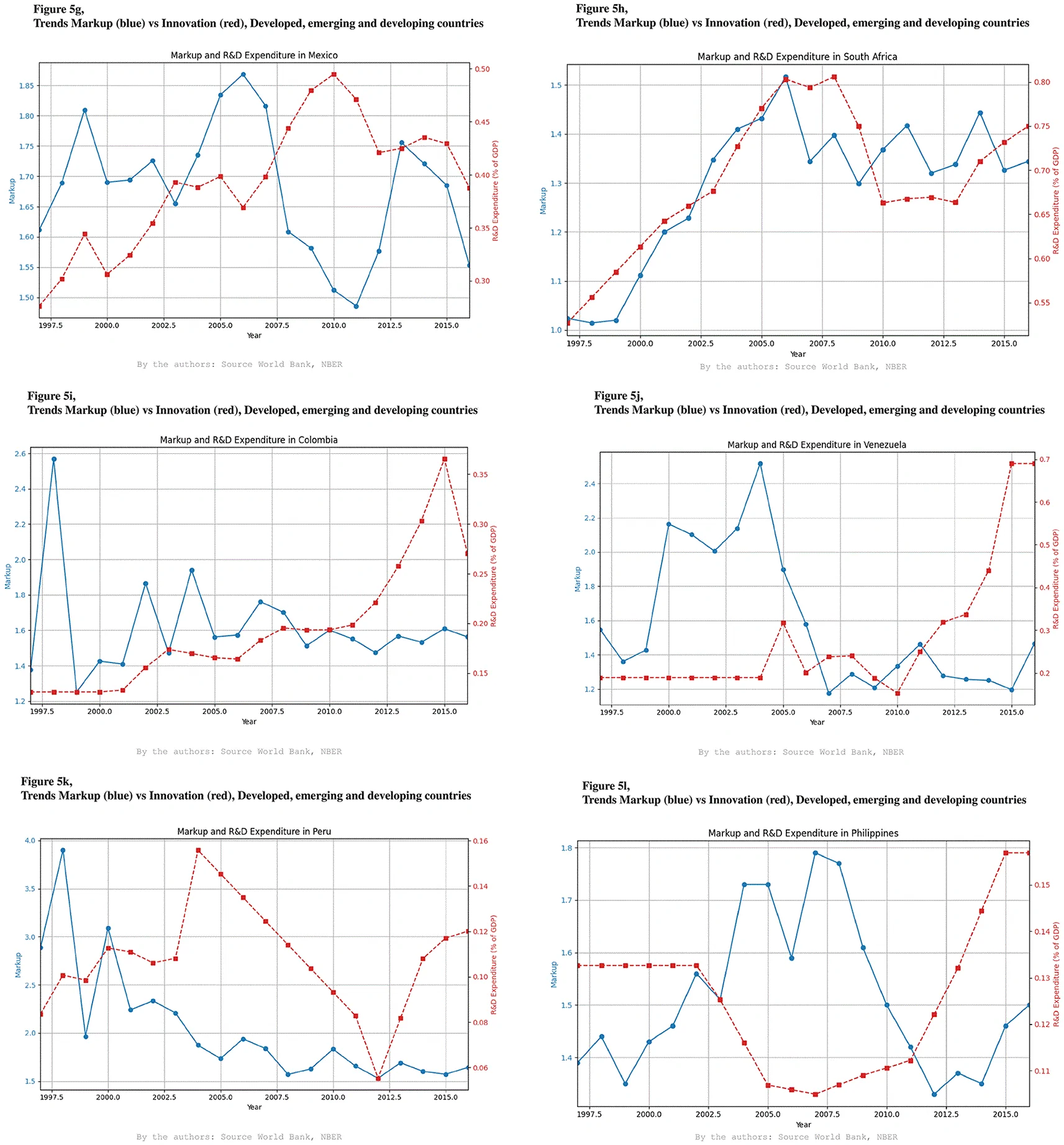
Trends Markup (blue) vs Innovation (red), Developed, emerging and developing countries.

Nevertheless, we must look at the bigger picture to understand what is happening. Other important factors can influence income distribution, such as taxes on income, GDP per capita (PPP), trade openness, poverty rates, institutional quality, and innovation as % of GDP:

By including these factors in a more detailed analysis, we can better understand how company profits interact with various elements of the economy and society to influence income inequality. It is a complex puzzle, and each piece helps us see how to promote a fairer distribution of wealth worldwide (  Figure 5).

### Trends markup vs innovation

There is a clear link in developed countries like the United States, Germany, and Japan: as these countries spend more on R&D, companies tend to make higher profits (measured by the markup). For example, in the United States, R&D spending increased from 2.48% of GDP in 1997 to 2.85% in 2016. At the same time, the average markup of companies went up from 1.55 to 1.78. This suggests that investing in R&D helps companies innovate and create better products or services, which allows them to charge more and increase their profits.

The relationship is more complex in emerging economies such as China, India, and Mexico. China significantly boosted its R&D spending from 0.64% to 2.10% of GDP between 1997 and 2016. However, the average company markup slightly increased from 1.30 to 1.40. This could mean that even though companies invest more in R&D, they face more competition, making it harder to raise prices and profits. The connection between R&D spending and company profits could be more explicit in India and Mexico, with ups and downs. This might be due to other factors like changes in government policies, economic reforms, or the level of competition in the market.

The link between R&D spending and company profits could be more apparent in developing countries like Peru, Colombia, and the Philippines. These countries spend less on R&D—often less than 0.5% of their GDP. For instance, Peru saw a slight increase in R&D spending but a significant drop in company markups from 2.88 to 1.64 between 1997 and 2016. This suggests that other factors, such as market structure, access to technology, or economic policies, might impact company profits more than R&D spending in these countries.

While richer countries tend to see a positive relationship between investing in R&D and company profits—likely because innovation leads to better products and higher prices—the story is more complicated in emerging and developing economies. In these places, other factors seem to play a more significant role in determining how much profit companies can make. To understand what is going on, we would need to look more closely at market competition, government policies, and how open the economy is to trade.

Given the clear heterogeneity observed among country groups, we acknowledge that using a unified empirical model might overlook key differences. However, our choice of a single model provides a generalized understanding, while suggesting future segmented analyses for deeper insights.

### Building the markup/income inequality model

First, we conduct a correlation analysis to mathematically determine which of the eight variables is most strongly correlated, both positively and negatively ( Table 3), and we see that the Gini index, a key measure of inequality, tells an important story about how economies function. A moderate negative correlation with GDP per capita (r = -0.33) suggests that the average income tends to be lower in countries where inequality is higher. This aligns with the idea that inequality limits opportunities and resource access, making it harder for economies to grow inclusively. A stronger negative correlation with net barter terms of trade (r = -0.73) shows that unequal countries often face more arduous trade conditions, possibly because they rely more on exporting lower-value goods, which might not benefit their economies significantly.

**Table 3.** Economic Indicators Dataset: Gini Index, GDP per Capita, Trade, Poverty, R&D, Taxes, and Markup.

	Gini Index	GDP per Capita (PPP)	Net Barter Terms of Trade	Poverty Headcount Ratio	R&D Expenditure	Taxes on Income	GDP	Markup
Gini Index	1.0	-0.33	-0.73	-0.16	-0.63	0.05	-0.22	0.61
GDP per Capita (PPP)	-0.33	1.0	0.04	-0.55	0.82	0.65	0.56	0.33
Net Barter Terms of Trade	-0.73	0.04	1.0	0.57	0.28	-0.05	0.07	-0.48
Poverty Headcount Ratio	-0.16	-0.55	0.57	1.0	-0.49	-0.12	-0.38	-0.38
R&D Expenditure	-0.63	0.82	0.28	-0.49	1.0	0.42	0.74	-0.04
Taxes on Income	0.05	0.65	-0.05	-0.12	0.42	1.0	0.69	0.67
GDP	-0.22	0.56	0.07	-0.38	0.74	0.69	1.0	0.38
Markup	0.61	0.33	-0.48	-0.38	-0.04	0.67	0.38	1.0

By the authors.

Looking at how inequality relates to other areas, the Gini index has a clear link to innovation. Countries with more inequality tend to spend less on research and development (r = -0.63), potentially creating a cycle where limited innovation holds back progress and worsens inequality. On the other hand, there is almost no connection between inequality and taxes on income, profits, and capital (r = 0.05), suggesting that tax systems alone might not address the problem of inequality. Interestingly, the Gini index does not strongly correlate with extreme poverty (r = -0.16), highlighting that inequality and poverty, while related, do not always move in the same direction.

An intriguing link emerges between the Gini index and markup, measuring how much firms can raise prices above costs (r = 0.61). This suggests that businesses may have greater market power in more unequal economies, which could further deepen disparities. Markup also has a weaker connection with GDP per capita (r = 0.33), hinting that wealthier countries do not necessarily have higher or lower markups. Its negative correlation with trade terms (r = -0.48) could mean that countries with less favourable trade deals rely more on domestic markets, where firms can exert more control over pricing. Interestingly, markup does not tie closely to innovation or poverty, showing that its drivers and effects are likely more specific to market structures than broader societal factors.

To achieve a more precise and comprehensive approach incorporating the variables considered in this study, we employed a two-way fixed-effects panel econometric model with country-level clustered standard errors. The model choice follows a diagnostic sequence rather than an assumption: Pesaran-type tests identify cross-sectional dependence, Wu-Hausman/Hausman tests favour fixed over random effects, and IPS/CADF-CIPS unit-root tests are used to evaluate whether long-run estimation in levels is appropriate. This framework analyses inequality drivers across diverse economic systems while acknowledging that cross-country dependence and non-stationarity affect inference.

When we run the econometric model, connecting all the variables, we see interesting patterns and relationships emerge. These insights help us understand how the variables interact and influence one another, shedding light on their roles and combined effects. We will progressively test each variable until we arrive at the final model:

***Equation***

$$\mathrm{Gin}i_{\left\{\mathrm{it}\right\}}=\alpha +\beta_{1}\cdot \text{MarkLa}g_{\left\{\mathrm{it}\right\}}+\beta_{2}\cdot \mathrm{Inn}o_{\left\{\mathrm{it}\right\}}+\beta_{3}\cdot \mathrm{Ta}x_{\left\{\mathrm{it}\right\}}+\beta_{4}\cdot \left(\text{MarkLa}g_{\left\{\mathrm{it}\right\}}\times \mathrm{Inn}o_{\left\{\mathrm{it}\right\}}\right)+\beta_{5}\cdot \text{Trad}e_{\left\{\mathrm{it}\right\}}+\beta_{6}\cdot \text{GDPp}c_{\left\{\mathrm{it}\right\}}+\gamma_{i}+\lambda_{t}+\varepsilon_{\left\{\mathrm{it}\right\}}$$

The contemporaneous markup term was tested during model development but is not retained in the preferred specification because the theoretical channel is delayed: market power affects inequality through pricing, wage bargaining, profit distribution and investment decisions that typically appear with a lag. The lag also reduces simultaneity between inequality and current markups. The interaction is limited to MarkLag×Innovation because the paper’s theoretical question concerns whether innovation amplifies or weakens the delayed effect of market power; additional interactions were explored as robustness checks but were not retained because they weakened parsimony without improving fit or theoretical clarity.

The primary goal was to understand the relationship between markups (aggregate profit margins) and inequality (measured by the Gini index), controlling for other relevant variables. The motivation arises from the idea that greater market power (reflected in high markups) could affect income distribution. Additionally, we included variables such as innovation, taxes on income, trade openness, per capita GDP, and, to a lesser extent, the economic system and poverty to isolate the impact of markups on inequality.

### Evolution of the analysis and modelling

The development of our model followed a structured progression to enhance its explanatory power and theoretical alignment:

1) **Basic Model: Gini ~ Mark**
  The initial model included only markups (mark) as the explanatory variable for inequality. While markup showed significance in this straightforward setup, the model lacked essential controls and omitted temporal dynamics, limiting its capacity for robust causal inference or thorough validation.
2) **Inclusion of controls and fixed effects**
  We expanded the model by incorporating key variables such as per capita GDP (gdpp), taxes (tax), poverty (pov), innovation (ino), trade openness (trade), and classifications of countries by development level or economic systems. Using a panel data approach with two-way fixed effects (country and year) and cluster-robust standard errors at the country level, we addressed heteroskedasticity and serial correlation issues, ensuring a more reliable framework.
3) **Introducing lagged markups (mark_lag)**
  As the inclusion of additional controls diminished the significance of the contemporaneous markup variable, we treated the effect of market power on inequality as a delayed process. We introduced mark_lag (markups lagged by one period) to capture the time required for pricing power to pass through to wages, consumer prices, profit shares and household income distribution. This choice is theoretically motivated and also reduces potential reverse causality between inequality and current markups. The contemporaneous markup term was examined during model development but was not retained because it did not materially improve the specification once the lagged term and fixed effects were included.
4) **Testing additional variables and refining the model**
  • **Poverty (pov) and GDP (GDP)**: While poverty was initially considered, it became insignificant when GDP per capita and other controls were included. Since GDP was significant and economically meaningful—suggesting that higher GDP per capita correlates with greater inequality due to uneven growth—it was retained, and POV was excluded.
  • **Innovation (ino) and interaction with mark_lag**: Innovation is retained because it may either diffuse productivity gains or reinforce market power when innovation rents accrue to leading firms. The interaction term I (mark_lag × ino) is therefore theoretically motivated: it tests whether innovation conditions the delayed inequality effect of market power. Other cross-products, including markup with trade and markup with taxation, were considered as robustness checks but were not retained in the main model because they did not add explanatory power and would over-parameterise a 12-country panel.
  • **Trade openness (trade)**: Trade emerged as significant and negatively associated with inequality. This finding suggests that greater international integration reduces income disparities, likely by fostering competition and curbing market power.

This iterative refinement process led to a theoretically grounded preferred model. We avoid describing it as definitively best, because the diagnostic results show that cross-sectional dependence, mixed integration order and cointegration affect how the estimates should be interpreted.

**Model rationale**

**Lagged markup (** $\text{MarkLa}g_{\left\{\mathrm{it}\right\}}$ **)**:

Captures the delayed impact of firms’ market power on income distribution, reflecting adjustments in the economy over time.

**Innovation (** $\mathrm{Inn}o_{\left\{\mathrm{it}\right\}}$ **)**:

It represents technological advancements that drive productivity and economic growth but may contribute to inequality by favouring skilled labour.

**Taxes on income (** $\mathrm{Ta}x_{\left\{\mathrm{it}\right\}}$ **)**:

Indicates redistributive fiscal policies to mitigate income inequality through progressive taxation and social programs.

**Trade openness (** $\text{Trad}e_{\left\{\mathrm{it}\right\}}$ **)**:

Measures international economic integration, which can reduce inequality by fostering competition and economic opportunities.

**GDP per capita (** $\text{GDPp}c_{\left\{\mathrm{it}\right\}}$ **)**:

It serves as a proxy for economic growth, acknowledging that higher GDP may not guarantee equitable income distribution.

**Interaction term (** $\text{MarkLa}g_{\left\{\mathrm{it}\right\}}\times \mathrm{Inn}o_{\left\{\mathrm{it}\right\}}$ **)**:

Explores the moderating role of innovation in the relationship between markups and inequality.

**Fixed effects (** $\gamma_{i}\;\text{and}\;\lambda_{t}$ **)**:

Fixed effects control for unobserved, time-invariant heterogeneity across units, allowing for unbiased estimation of the variables of interest.

## Key findings

The chosen model employs a panel with two-way fixed effects (country and year) and cluster-robust standard errors at the country level. The analysis highlights key drivers of inequality (  Table 4). Lagged markups (mark_lag) significantly increase inequality, while innovation (ino) shows a weak, non-significant reduction. Tax revenue (tax) strongly reduces inequality, emphasising its redistributive role. The interaction between markups and innovation (I (mark_lag * ino)) is positive but insignificant, requiring further investigation. Trade openness (trade) significantly reduces inequality, whereas GDP per capita (gdpp) slightly increases it. Suggesting that growth alone may not address inequality effectively, reflecting the complex interplay of economic and policy factors shaping income disparities.

**Table 4.** Regression analysis results.

Metric	Value	Interpretation
Residuals		
Minimum	-2.583748	Indicates the most significant negative deviation between observed and predicted Gini Index values.
1st Quartile	-0.740136	Represents the lower 25% of residuals.
Median	0.064233	A median residual close to zero indicates a well-centred model.
3rd Quartile	0.524138	Represents the upper 25% of residuals.
Maximum	3.040091	Indicates the most significant positive deviation between observed and predicted Gini Index values.
Coefficients		
mark_lag	1.9161 (p = 0.0087)	Significant positive relationship: Higher markups (with a lag) are associated with higher inequality (Gini Index).
ino	-3.0699 (p = 0.2884)	Negative but not significant, suggesting no strong evidence that innovation reduces inequality in this model.
tax	-0.2463 (p = 9.053e-07)	Highly significant negative relationship: increasing tax revenue reduces inequality, reflecting strong redistributive power.
I (mark_lag * ino)	2.5642 (p = 0.1058)	Positive but insignificant: The interaction between lagged markups and innovation has no strong effect on inequality.
trade	-0.0399 (p = 0.0015)	Significant negative relationship: Higher trade openness is associated with lower inequality.
gdpp	0.0003 (p = 8.621e-05)	Significant positive relationship: higher GDP per capita is associated with slightly higher inequality.
Model Fit		
Total Sum of Squares	272.49	Total variability in the Gini Index within the dataset.
Residual Sum of Squares	82.234	Variability in the Gini Index is unexplained by the model.
R-Squared	0.69821	Indicates that the model explains 69.8% of the variability in inequality.
Adjusted R-Squared	0.50461	Adjusted for degrees of freedom, the model still explains 50.4% of inequality variation.
F-statistic	142.958 (p = 4.1866e-09)	Highly significant, confirming that the overall model is statistically robust and valid.

By the authors.

Why this specification is retained as the preferred model

• Stability Across Specifications: Key variables like mark_lag and tax remain robustly significant across various specifications, reinforcing their reliability. The lagged markup (t−1) is included to capture persistent effects of market power and mitigate endogeneity concerns, as contemporaneous markups (t) may correlate with unobserved shocks. The innovation-markup interaction at t−1 reflects theoretical expectations of delayed impacts from innovation on pricing strategies. Additional cross-terms were excluded to avoid overfitting and maintain parsimony, guided by empirical tests showing no significant improvement in model fit.
• Captures Lagged Effects: The inclusion of mark_lag highlights how market power impacts inequality over time, a relationship that is not evident in contemporaneous models.
• Alignment with Theory: The findings are consistent with existing research—progressive taxes help reduce inequality, trade openness fosters competition to narrow income gaps, and GDP growth, without inclusivity, risks exacerbating disparities.
• Strong Explanatory Power, with diagnostic caution: With an R-squared close to 70% and a highly significant F-statistic, the model captures a large proportion of the variability in inequality. However, this fit statistic should be read alongside the panel diagnostics, since cross-sectional dependence and non-stationarity can affect standard inference.

This model is selected as the preferred specification because it aligns with the theoretical timing of market-power effects and is supported by the diagnostic sequence reported below. The word “preferred” is used deliberately: the specification is not presented as the only valid model, but as the most defensible baseline after testing for cross-sectional dependence, fixed-versus-random effects, unit roots and cointegration. Dynamic panel and error-correction approaches remain useful extensions for future work.

The first diagnostic step examined cross-sectional dependence. Pesaran’s scaled LM test, with the Pesaran, Ullah and Yamagata (2008) correction, detects significant residual dependence (p < 0.05), implying that shocks in one country may reverberate across the 12-country panel. This result does not invalidate the fixed-effects design, but it means inference should be interpreted with robust or common-factor-aware standard errors. The second step compared random and fixed effects. The Wu-Hausman/Hausman test rejects the random-effects null (p < 0.01), confirming that unobserved country heterogeneity is correlated with the regressors and that a fixed-effects framework is more suitable than random effects for the baseline specification.

As a robustness check, the fixed-effects model was re-estimated with common-correlated-effects terms to account for detected dependence, after applying the documented missing-value rules for scattered observations in trade, innovation and GDP per capita. The qualitative story holds, although magnitudes are attenuated. This pattern supports the central association between higher lagged markups and higher inequality, while also showing why the coefficient should be interpreted as a conditional long-run association rather than as a mechanically causal short-run effect.

The third diagnostic step examined unit roots. The Im, Pesaran and Shin (2003) test was used as a first-generation panel unit-root test, while Pesaran’s (2007) CADF/CIPS approach was used to allow for cross-sectional dependence. This sequence is important because the IPS test assumes no contemporaneous correlation and may overstate stationarity when countries share global shocks. The results indicate mixed integration orders: some core series, including markups and GDP per capita, behave as I(1), while others are closer to stationary. This mixed evidence requires cointegration testing before interpreting the levels specification as a long-run model.

The fourth diagnostic step tested cointegration. Given the mixed integration order, Westerlund’s (2007) panel cointegration test was applied because it is robust to serial correlation and can be implemented with cross-sectional dependence in mind. The group statistics provide partial evidence of a long-run equilibrium relationship among the variables. This supports estimating the model in levels as a long-run association, but it also requires caution: if cointegration were absent, the levels model could be inefficient or spurious. For this reason, the results are interpreted as long-run conditional associations and are complemented by first-difference and error-correction checks.

Accordingly, we re-estimated the model using both first-difference and error-correction approaches. In the first-differenced specification, the lagged markup coefficient decreased and lost statistical significance, which is expected if the main effect operates through a long-run channel rather than a short-run annual change. In the error-correction model, the long-run coefficient for lagged markups remained positive and significant, while the negative adjustment coefficient indicated convergence toward equilibrium. These checks reinforce the main finding that higher markups are associated with greater income inequality over time, provided the relationship is interpreted through a long-run panel framework.

## Policies recommendations

Given the significant lagged effects of markup trends, competition authorities should closely monitor market structures and pricing power. Encouraging fair competition through reduced entry barriers, more vigorous enforcement against anti-competitive practices, and ongoing market oversight can mitigate inequality over time, particularly in emerging and developing countries.

Prioritising progressive and efficient tax systems is essential to effectively addressing inequality. Strengthening tax collection mechanisms and channelling resources into well-targeted social programs can maximise the redistributive impact.

The link between per capita GDP and inequality highlights that economic growth alone is insufficient for equitable outcomes. Policies that expand access to education provide robust social safety nets and ensure fair labour market practices are critical to distributing growth’s benefits more evenly.

Finally, the negative correlation between trade and inequality underscores the potential of global integration to narrow income gaps. However, to achieve inclusive benefits, complementary measures are needed to support vulnerable sectors, encourage technology adoption, and protect groups at risk of being left behind.

## Conclusion

Our analysis provides cross-country empirical evidence that increasing market power, measured through higher lagged markups, is associated with wider income inequality, with the relationship particularly relevant in emerging and developing economies where institutional quality and redistributive capacity differ from advanced economies ( Auray et al., 2020; Eeckhout et al., 2024). The revised diagnostics strengthen the methodological basis of the preferred specification: cross-sectional dependence is present, fixed effects are favoured over random effects, several variables show non-stationary behaviour, and Westerlund-style tests provide partial evidence of cointegration. These results support a long-run interpretation while also limiting the strength of causal claims.

The preferred model, which includes mark_lag and key control variables (innovation, taxation, GDP per capita and trade openness) with two-way fixed effects, offers a defensible explanation of how delayed market power and fiscal policy relate to inequality. By controlling for country-specific and year-specific effects, the model captures structural differences between more developed economies, where fiscal policies and trade openness tend to have stronger redistributive effects, and less developed economies, where market power and weaker institutions may exacerbate inequality. The evidence supports context-specific policy responses that combine fiscal redistribution, active competition enforcement and institutional reform, especially in emerging and developing settings. The findings should nevertheless be read as conditional long-run associations rather than as fully settled causal estimates.

## Ethics and consent

No ethics and consent were required.

## Data Availability

The dataset used in this study is publicly available and sourced from reputable organizations, including the **World Bank** and the **National Bureau of Economic Research (NBER)**. All data can be accessed through their official platforms using the same methods as the authors. Detailed instructions and links for accessing the datasets are provided to ensure readers and reviewers can replicate the analysis and apply the methodology described in this article. Additionally, any supplementary or representative data required for applying the methodology are also publicly accessible and included for reference. All necessary information required for a reader or reviewer to access the data by the same means as the authors. The data is primarily sourced from the World Bank database ( https://databank.worldbank.org), where users can filter by variable and period. Data on markups, however, comes exclusively from the National Bureau of Economic Research (NBER) database, based on De Loecker and Eeckhout’s (2017) study, *“The Rise of Market Power and the Macroeconomic Implications”* (NBER working paper). The dataset, titled *“Aggregate Markups from Global Market Power: Country-year (xls) and Continent-year (xls),”* is available at https://sites.google.com/site/deloeckerjan/data-and-code, providing essential insights for macroeconomic analyses.

## Footnotes

^1^
Extractive institutions: According to Acemoglu, Johnson, and Robinson (Nobel Prize winners in economics), extractive institutions are structures that concentrate economic and political power in the hands of an elite, allowing them to extract wealth from most of the population. These institutions limit innovation, competition, and economic growth, favouring exploitation and perpetuating inequality. Unlike inclusive institutions, which encourage participation and development, extractive institutions generate political instability and long-term poverty. Examples include colonialism and authoritarian regimes.

^2^
Developed Countries are typically associated with free-market economies characterised by strong institutional frameworks, robust regulatory systems, and market-oriented dynamics. Emerging Economies Tend to have hybrid economic systems, combining market elements with a significant role of the state in resource management and policymaking. Developing Countries Often operate under mixed or state-influenced systems, with weaker institutions and regulatory challenges. By classifying by level of development, a significant portion of the dynamics of economic systems is already being captured ( Todaro and Smith, 2020).

## References

[ref1] Acemoglu D Robinson JA : Weak, despotic, or inclusive? How state type emerges from state versus civil society competition. *Am. Polit. Sci. Rev.* 2023;117(2):407–420.

[ref3] Aghion P Akcigit U Bergeaud A : Innovation and top income inequality. *Rev. Econ. Stud.* 2019;86(1):1–45. 10.1093/restud/rdy027

[ref4] Aghion P Antonin C Bunel S : *The power of creative destruction: Economic upheaval and the wealth of nations.* Harvard University Press;2021.

[ref5] Almunia M Antràs P : Markups and inequality in emerging markets. *Econ. Stud. J.* 2019.

[ref6] Atkinson AB : *Inequality: What can be done?* Harvard University Press;2015. 10.4159/9780674287013

[ref62] Auray S Eyquem A Gauthier B : *Markups, Taxes, and Rising Inequality.* Aix-Marseille School of Economics Working Papers;2020.

[ref7] Autor D Dorn D Katz LF : The fall of the labour share and the rise of superstar firms. *Q. J. Econ.* 2020;135(2):645–709. 10.1093/qje/qjaa004

[ref59] Bakir E Hays M Knoedler J : Rising Corporate Power and Declining Labor Share in the Era of Chicago School Antitrust. *J. Econ. Issues.* 2021;55(2):397–407. 10.1080/00213624.2021.1908802

[ref8] Barr N : *Economics of the welfare state.* USA: Oxford University Press;2020.

[ref9] Carlton DW Perloff JM : *Modern industrial organisation.* 4th ed. Pearson;2005.

[ref56] CEIC Data: *Australia - Gini Coefficient.* CEIC;2024. Retrieved October 14, 2024. Reference Source

[ref10] Chang H-J : *Bad Samaritans: The myth of free trade and the secret history of capitalism.* Bloomsbury Press;2008.

[ref61] Colciago A Mechelli R : *Competition and Inequality.* Department of Economics Discussion Paper Series: University of Oxford;2020. 10.2139/ssrn.3652023

[ref11] Collier P : *The bottom billion: Why the poorest countries are failing and what can be done about it.* Oxford University Press;2007.

[ref12] De Loecker J Eeckhout J : *Global market power.* National Bureau of Economic Research;2018. Aggregate markups from Global Market Power. Reference Source

[ref13] Demir A Pesque-Cela V Altunbas Y : FinTech, financial inclusion and income inequality: A quantile regression approach. 2022. 10.1080/1351847X.2020.1772335

[ref14] Easterly W : *The Elusive Quest for Growth: Economists’ Adventures and Misadventures in the Tropics.* MIT Press;2001.

[ref63] Eeckhout J Fu C Li W : Optimal Taxation and Market Power. *SSRN Electronic Journal.* 2024. 10.2139/ssrn.4770758

[ref15] Enke S : Consumer coöperatives and economic efficiency. *Am. Econ. Rev.* 1945;148–155.

[ref16] Fehr E Schmidt KM : A theory of fairness, competition, and cooperation. *Q. J. Econ.* 1999;114(3):817–868. 10.1162/003355399556151

[ref18] Friedman M : *Capitalism and freedom.* University of Chicago Press;1962.

[ref19] Galbraith JK : *The affluent society.* Houghton Mifflin;1998.

[ref20] Guellec D Paunov C : *Digital innovation and the distribution of income.* National Bureau of Economic Research;2017. Reference Source

[ref21] Gunasinghe C Selvanathan EA Naranpanawa A : Rising income inequality in OECD countries: Does fiscal policy sacrifice economic growth in achieving equity? *Eur. J. Dev. Res.* 2021;33:1840–1876. 10.1057/s41287-020-00322-8

[ref58] Han M Pyun J : Markups and income inequality: Causal links, 1975-2011. *J. Comp. Econ.* 2021;49(2):290–312. 10.1016/j.jce.2020.12.002

[ref22] Harrell FE : *Regression modelling strategies: With applications to linear models, logistic regression, and survival analysis.* Springer;2015.

[ref23] Hovenkamp H : Worker welfare and antitrust. *Univ. Chic. Law Rev.* 2023;90(2):511–544.

[ref65] Im KS Pesaran MH Shin Y : Testing for unit roots in heterogeneous panels. *J. Econom.* 2003;115(1):53–74. 10.1016/S0304-4076(03)00092-7

[ref25] IMF: *World Economic Outlook.* Washington, D.C.: International Monetary Fund;2025. Reference Source

[ref26] Kahneman D : Maps of bounded rationality: A perspective on intuitive judgment and choice. *Nobel Prize Lecture.* 2002.

[ref27] Karagiannis N Tsoulfidis L : Innovation and income inequality: World evidence. *Munich Personal RePEc Archive.* 2019. Reference Source

[ref57] Lee H Lee J : Aggregate Markup and Its Impact on Income Inequality: Country Panel Evidence. *Int. Econ. J.* 2023;37(3):387–400. 10.1080/10168737.2023.2239204

[ref60] Lee H Lee J Yoon H : Accounting for the Effect of Government Spending on Income Inequality: Role of Markup Dynamics. *Korea World Econ.* 2021;22(3):129–157. 10.46665/kwe.2021.12.22.3.129

[ref28] Lin JY Chang H-J : Should Industrial Policy in Developing Countries Conform to Comparative Advantage or Defy It? *Dev. Policy Rev.* 2009;27(5):483–502. 10.1111/j.1467-7679.2009.00456.x

[ref29] Maganya MH : Tax revenue and economic growth in a developing country: an autoregressive distribution lags approach. *Cent. Eur. Econ. J.* 2020;7(54):205–217. 10.2478/ceej-2020-0018

[ref30] McCullagh P Nelder JA : *Generalised linear models.* 2nd ed. Chapman & Hall;1989.

[ref31] Monga C Ndulu B : *The Oxford Handbook of Africa and Economics.* Oxford University Press;2020.

[ref32] Moon S Jambert E Childs M : A win-win solution?: A critical analysis of tiered pricing to improve access to medicines in developing countries. *Glob. Health.* 2011;7:11–39. 10.1186/1744-8603-7-39 PMC3214768 21992405

[ref34] North DC : Institutions. *J. Econ. Perspect.* 1991;5(1):97–112. 10.1257/jep.5.1.97

[ref33] North DC : *Institutions, institutional change, and economic performance.* Cambridge University Press;1990.

[ref35] OECD: *In it together: Why less inequality benefits all.* OECD Publishing;2015.

[ref36] Olson M : *The rise and decline of nations: Economic growth, stagflation, and social rigidities.* Yale University Press;1982.

[ref37] Pacheco-Jaramillo WAP : *Understanding Subjective Well-being, Prosocial Activities and the Sumak-Kawsay “The Good Way of Living”: An Ecuadorian Case Study.* University of Canberra;2023.

[ref67] Pesaran MH : A simple panel unit root test in the presence of cross-section dependence. *J. Appl. Econom.* 2007;22(2):265–312. 10.1002/jae.951

[ref70] Pesaran MH Ullah A Yamagata T : A bias-adjusted LM test of error cross-section independence. *Econometrics Journal.* 2008;11(1):105–127. 10.1111/j.1368-423X.2007.00227.x

[ref38] Piketty T : *Capital in the twenty-first century.* Harvard University Press;2014.

[ref55] Robinson JA Acemoglu D : *Why nations fail: The origins of power, prosperity and poverty.* London: Profile;2012; pp.45–47.

[ref39] Rodrik D : *Industrial Policy for the Twenty-First Century.* Harvard University;2004. KSG Working Paper No. RWP04-047. 10.2139/ssrn.617544

[ref40] Rybakov AV Shichkin IA Tolmachev OM : The impact of a progressive personal income tax scale on reducing income inequality: Comparative analysis. *Relacoes Internacionais no Mundo Atual.* 2022;1(34):371–395.

[ref41] Sachs JD : *The end of poverty: Economic possibilities for our time.* Penguin Press;2005. 10.1111/j.1600-0579.2007.00476.x 18289264

[ref42] Schmalensee R : Interindustry studies of structure and performance. Schmalensee R Willig RD , editors. *Handbook of industrial organisation.* Vol.1. North-Holland;1989; pp.951–1009.

[ref43] Sen A : *Development as freedom.* Oxford University Press;1999.

[ref44] Spulber DF : Antitrust and innovation competition. *J. Antitrust Enforc.* 2023;11(1):5–50. 10.1093/jaenfo/jnac013

[ref45] Statista: Gender pay gap index in selected Latin America and the Caribbean countries in 2023. 2023. Reference Source

[ref46] Stigler GJ : *The organisation of industry.* University of Chicago Press;1988.

[ref47] Stiglitz JE : *Globalisation and its discontents.* W.W. Norton & Company;2001.

[ref49] Stiglitz JE : *People, power, and profits: Progressive capitalism for an age of discontent.* W.W. Norton & Company;2020.

[ref48] Stiglitz JE : *The price of inequality: How today’s divided society endangers our future.* W.W. Norton & Company;2012.

[ref54] Syverson C : Macroeconomics and market power: Context, implications, and open questions. *J. Econ. Perspect.* 2019;33(3):23–43. 10.1093/jaenfo/jnac013

[ref50] Thaler RH : *Misbehaving: The making of behavioural economics.* W.W. Norton & Company;2017.

[ref51] Tirole J : *The theory of industrial organisation.* MIT Press;1988.

[ref52] Todaro MP Smith SC : *Economic Development.* 13th ed. Pearson Education;2020.

[ref53] Tybout JR : Manufacturing firms in developing countries: How well do they do, and why? *J. Econ. Lit.* 2000;38(1):11–44. 10.1257/jel.38.1.11

[ref66] Westerlund J : Testing for error correction in panel data. *Oxf. Bull. Econ. Stat.* 2007;69(6):709–748. 10.1111/j.1468-0084.2007.00477.x

