# Supplementary material for: Cross-country analysis of aggregate markups and their impact on income inequality
Source: F1000Res. 2026 Aug 27;14:184. Originally published 2025 Feb 10. [Version 4] doi: 10.12688/f1000research.160674.4 (PMC13597091; doi:10.12688/f1000research.160674.4)
Supplement: Peer review history

# Reviewer report: Reviewer response for version 4

**DOI:** 10.5256/f1000research.194943.r516245

**Published:** 2026-09-01

**Authors:** Thanh Huu Phu Nguyen

**Affiliations:** 1 Department of Finance and Accounting Management, Ho Chi Minh City University of Law, Ho Chi Minh City, Vietnam

The manuscript investigates the relationship between aggregate markups and income inequality using panel data for 12 countries over 1997–2016. The authors employ a two-way fixed-effects model and, in the revised version, add tests for cross-sectional dependence, fixed versus random effects, unit roots, cointegration, and several robustness checks. The topic is relevant and the revised methodological discussion is substantially improved. The main finding is that higher lagged markups are associated with higher income inequality, while taxation and trade openness are negatively associated with inequality. 

 However, several issues should be addressed before the article can be considered fully sound.

 First, the manuscript should distinguish more carefully between  **markups, profits, and profit margins**. The markup variable is defined as the ratio of price to marginal cost, but several parts of the text interpret it as company profit or profit margin. These concepts are not equivalent, and the terminology should be made consistent throughout.

 Second, the definition of the  **trade variable** is unclear. The empirical model refers to trade openness, while the correlation table discusses “Net Barter Terms of Trade.” The authors should clearly state the exact indicator, source, unit of measurement, and which variable is included in the final regression.

 Third, the  **tax variable** should be interpreted more cautiously. The measure used is the share of taxes on income, profits, and capital gains in total revenue, which is not a direct measure of tax progressivity. Therefore, conclusions regarding progressive taxation and redistribution should be softened unless additional evidence is provided.

 Fourth, the statistical results need more transparent reporting. The manuscript discusses Pesaran tests, Hausman tests, unit-root tests, Westerlund cointegration tests, common-correlated-effects estimates, first-difference estimates, and an error-correction specification, but many of these results are only described narratively. A table reporting test statistics, exact p-values, coefficients, standard errors, sample sizes, and key robustness estimates would materially improve reproducibility and evaluation of the results.

 Fifth, the claim that the relationship between markups and inequality is particularly strong in emerging and developing economies is not directly established by the preferred pooled fixed-effects model. The current specification estimates a common markup coefficient across countries. The authors should either estimate group-specific effects or interactions between markups and development groups, or substantially soften this conclusion.

 Finally, the manuscript would benefit from careful proofreading. Some sentences appear to contain residual reviewer or editorial comments embedded in the text and should be removed before indexing. 

 The data sources are publicly identified and appear accessible through the World Bank and the NBER markup dataset, which supports reproducibility in principle.

Is the work clearly and accurately presented and does it cite the current literature?

Partly

If applicable, is the statistical analysis and its interpretation appropriate?

Partly

Are all the source data underlying the results available to ensure full reproducibility?

Yes

Is the study design appropriate and is the work technically sound?

Partly

Are the conclusions drawn adequately supported by the results?

Partly

Are sufficient details of methods and analysis provided to allow replication by others?

Partly

Reviewer Expertise:

Economics

I confirm that I have read this submission and believe that I have an appropriate level of expertise to confirm that it is of an acceptable scientific standard, however I have significant reservations, as outlined above.

# Reviewer report: Reviewer response for version 3

**DOI:** 10.5256/f1000research.180931.r383948

**Published:** 2025-06-02

**Authors:** Anna Maria Grazia Variato

**Affiliations:** 1 Università degli Studi di Bergamo, Bergamo, Italy

Dear Editor and Authors,

 I went through this third version of the paper and in my opinion the revisions are sound and enough to make the paper indexable.

 I appreciate the effort made by the authors.

Is the work clearly and accurately presented and does it cite the current literature?

Partly

If applicable, is the statistical analysis and its interpretation appropriate?

Partly

Are all the source data underlying the results available to ensure full reproducibility?

Yes

Is the study design appropriate and is the work technically sound?

Partly

Are the conclusions drawn adequately supported by the results?

Partly

Are sufficient details of methods and analysis provided to allow replication by others?

Yes

Reviewer Expertise:

Macroeconomic modelling under limited information and heterogeneity, Financial Instability, Financial sustainability and Inequality, growth cycles (or AD led growth models)

I confirm that I have read this submission and believe that I have an appropriate level of expertise to confirm that it is of an acceptable scientific standard.

# Reviewer report: Reviewer response for version 2

**DOI:** 10.5256/f1000research.180368.r380034

**Published:** 2025-05-16

**Authors:** Anna Maria Grazia Variato

**Affiliations:** 1 Università degli Studi di Bergamo, Bergamo, Italy

I went through the new version of the paper and I saw the improvements made. In my opinion this is enough for the paper to be accepted.

 I am glad that my suggestions were somehow helpful.

 Just a minor issue: in the introduction the author uses first person, but I understood that the paper has two authors so I expected "we".

Is the work clearly and accurately presented and does it cite the current literature?

Partly

If applicable, is the statistical analysis and its interpretation appropriate?

Partly

Are all the source data underlying the results available to ensure full reproducibility?

Yes

Is the study design appropriate and is the work technically sound?

Partly

Are the conclusions drawn adequately supported by the results?

Partly

Are sufficient details of methods and analysis provided to allow replication by others?

Yes

Reviewer Expertise:

Macroeconomic modelling under limited information and heterogeneity, Financial Instability, Financial sustainability and Inequality, growth cycles (or AD led growth models)

I confirm that I have read this submission and believe that I have an appropriate level of expertise to confirm that it is of an acceptable scientific standard.

# Response

**Published:** 2025-05-26

**Authors:** William Pacheco

**Affiliations:** Economics, University Anahuac Mexico, Huixquilucan de Degollado, State of Mexico, Mexico

Thank you very much for your time and for confirming the scientific standard of the revised submission. I truly appreciate your valuable feedback throughout the process.

# Reviewer report: Reviewer response for version 1

**DOI:** 10.5256/f1000research.176605.r367355

**Published:** 2025-03-19

**Authors:** Chrysovalantis Amountzias

**Affiliations:** 1 University of Hertfordshire, Hertfordshire, UK

The current paper investigates the relationship between markups and income inequality, when certain control variables are taken into consideration. By using a Fixed effects model with both individual and time effects, the authors find that higher markups are closely linked to greater income inequality, with the impact being particularly pronounced in emerging and developing economies. The presentation of data and the analysis of relationships between the Gini coefficient and the De Loecker and Eeckhout markup ratios is interesting, but there are a few methodological points that need to be addressed:

1. The authors use a fixed effects approach, due to the presence of correlation between the individual effects and the explanatory variables. As I suspect that could be the case, the authors do not provide any diagnostic tests pointing to this outcome. Therefore, I strongly recommend using Pesaran’s scaled (LM) test (Pesaran, 2004) as corrected by Pesaran, Ullah and Yamagata (2008), given that this test identifies the presence of cross-sectional dependence across the panel entities and thus, whether a random effects model is more suitable compared to the pooled OLS estimation technique without any individual effects. After that, the test developed by Wu (1973) and Hausman (1978) should be used to check whether the random or the fixed effects model is more suitable.
2. A major omission from the methodological approach is the investigation of unit roots. As the panel set consists of 12 countries for 20 years, I suspect that non-stationarity may be present. In this case, the Im, Pesaran and Shin (2003) and Pesaran (2007) tests should be employed to identify the order of integration of the panel series when cross section dependency is taken into consideration. The former test assumes the absence of contemporaneous correlation and thus, it may not result in accurate results. For this reason, the latter test is also employed by using cross section ADF tests (CADF) where the initial ADF regression is augmented by the cross section average values of lagged levels and first order differences. If at least one of the series is found to be first order integrated, the presence of cointegration must be tested and conclude whether a long-run relationship in the model.
3. For cointegration, Westerlund’s (2007) test is a robust test for panel cointegration allowing for cross-sectional dependence and can be used when there are both serial correlation and cross-sectional dependence. If cointegration persists, the authors could use the fixed effects approach in their model, including stationary panel series.
4. In model formulation, the authors do not explain the theoretical background for choosing some of their variables. For instance, the lagged value of the markup ratio is included, but the variable at time *t* is absent. Why is this case? The reader would be expecting to see the long run relationship between the dependent and the explanatory variable at time *t*. Moreover, there is no explanation of why there is only a cross-product between innovation and markup at time *t-1*. Why are additional cross-products not included? Is this an empirical issue or does it depend on theoretical grounds? The authors should make this crystal clear.
5. In page 18, the authors state “This model is considered the best based on the following reasons”. Although their reasons are contributing to the clarity of the model, the issues raised in my previous points (1-4) clearly show that it is not the best alternative, as the presence of unit roots may render the model inefficient. Therefore, this statement is too strong and ambitious, given the current state of the model.

**References**

 [Ref 1-5]

Is the work clearly and accurately presented and does it cite the current literature?

Yes

If applicable, is the statistical analysis and its interpretation appropriate?

Partly

Are all the source data underlying the results available to ensure full reproducibility?

Yes

Is the study design appropriate and is the work technically sound?

Yes

Are the conclusions drawn adequately supported by the results?

Yes

Are sufficient details of methods and analysis provided to allow replication by others?

Yes

Reviewer Expertise:

Industrial Organisation, market analysis, pricing decisions, competitive strategies, income inequality.

I confirm that I have read this submission and believe that I have an appropriate level of expertise to confirm that it is of an acceptable scientific standard, however I have significant reservations, as outlined above.

## References

[1] : Specification Tests in Econometrics. *Econometrica* .1978;46(6) : 10.2307/1913827 10.2307/1913827

[2] : Testing for unit roots in heterogeneous panels. *Journal of Econometrics* .2003;115(1) : 10.1016/S0304-4076(03)00092-753-74 10.1016/S0304-4076(03)00092-7

[3] : Estimation and Inference in Large Heterogeneous Panels with a Multifactor Error Structure. *Econometrica* .2006;74(4) : 10.1111/j.1468-0262.2006.00692.x967-1012 10.1111/j.1468-0262.2006.00692.x

[4] : A simple panel unit root test in the presence of cross‐section dependence. *Journal of Applied Econometrics* .2007;22(2) : 10.1002/jae.951265-312 10.1002/jae.951

[5] : A bias‐adjusted LM test of error cross‐section independence. *The econometrics journal* . https://www.jstor.org/stable/23116064 10.1111/j.1368-423X.2007.00227.x

# Response

**Published:** 2025-04-20

**Authors:** William Pacheco

**Affiliations:** Economics, University Anahuac Mexico, Huixquilucan de Degollado, State of Mexico, Mexico

We sincerely thank the reviewer for their insightful methodological feedback. We have addressed cross-sectional dependence (Pesaran’s test), non-stationarity (IPS/CIPS), and cointegration (Westerlund’s test), while clarifying variable lag structures to align with theoretical persistence and endogeneity mitigation. These revisions significantly strengthen the robustness and transparency of our analysis.

# Response

**Published:** 2026-01-06

**Authors:** William Pacheco

**Affiliations:** Economics, University Anahuac Mexico, Huixquilucan de Degollado, State of Mexico, Mexico

Thank you very much for your valuable feedback. We have made the necessary revisions to address the points you raised. Please note that due to an oversight, I mistakenly sent the changes to Reviewer 1 instead of you. I apologize for any confusion caused, and I greatly appreciate your patience.

 Regarding your comments:

1. Pesaran’s Scaled (LM) Test and Random Effects Model:
  We have implemented Pesaran’s scaled (LM) test (Pesaran, 2004), as well as the corrections by Pesaran, Ullah, and Yamagata (2008), to detect cross-sectional dependence across the panel entities. Based on the test results, we have confirmed that a random effects model is not more suitable than the fixed effects model. We have updated the paper to reflect this change and have included the diagnostic tests as per your suggestion.
2. Unit Root Tests:
  We have conducted the Im, Pesaran, and Shin (2003) test, as well as the Pesaran (2007) test, to check for non-stationarity and potential unit roots. The results suggest that some of the series are indeed non-stationary, and we have accordingly tested for cointegration using Westerlund’s (2007) panel cointegration test. We have found evidence of cointegration, and the long-run relationship is now properly integrated into the model.
3. Methodological Approach and Model Formulation:
  Regarding the omission of the variable at time t and the cross-product between innovation and markup at time t-1, we have clarified these points in the revised manuscript. We have explained that the lagged value of the markup ratio was included to capture the dynamic nature of markups in relation to income inequality, while omitting the variable at time t due to theoretical considerations of its diminishing effect in our model. The inclusion of only the cross-product at t-1 is based on theoretical grounds, which we have now elaborated on in the revised version of the paper.
4. Statement on Model Suitability:
  We have rephrased the statement on page 18, acknowledging that while our model is the best available given the current data and methodological considerations, we recognize the potential inefficiencies due to the presence of unit roots. This statement has been revised to reflect a more balanced view of the model’s strengths and limitations.

We believe that the revisions have strengthened the methodological robustness of the paper, and we hope that the changes meet your expectations.

 Once again, thank you for your thoughtful and constructive comments. We look forward to receiving your feedback on the revised manuscript.

# Reviewer report: Reviewer response for version 1

**DOI:** 10.5256/f1000research.176605.r367356

**Published:** 2025-03-10

**Authors:** Anna Maria Grazia Variato

**Affiliations:** 1 Università degli Studi di Bergamo, Bergamo, Italy

I like the paper especially with respect to the background and the observation that the field focus of the paper needs to be explored and developed. Overall, I have a positive view of the project behind this study.

 There is overall coherence among the title of the research and the paper. However, the development of the argument throughout the sections could be refined, which leads me to suggest some revisions before considering acceptance.

 I hope my comments will be a useful contribution towards reaching a higher argumentative strength of this possible piece of economic literature.

 Comment 1: with respect to the first reserve on literature presented. The literature provided by authors is in general correctly stated, but it is not true that the research is fully novel. I have included at the end of this report a set of references that I believe will be useful to the authors. As a result, I would suggest changing the statement at page 3 paragraph 4 where authors say “…the impact of markups […] has been widely studied, their direct relationship with income inequality […] has yet to be explored.”

 In the introduction qualitative aspects (like excessively high markups), or behavioral aspects (like inequality aversion) are introduced, and linked to pertinent pieces of literature, but these pieces of literature are not functional to the development of the argument in the paper: none of the two elements becomes a determinant of the original empirical structure proposed by the authors… then what is the point to quote this literature?

 My suggestion in this respect is either to drop the passage, or to qualify the relevance of the observation in the introduction (which I do not object, but I consider too much for the length of just one paper) finalizing it in at least a comment in the section where the model is presented and estimated.

 In the theoretical framework section authors say “the link between markups and income inequality has been subject on intense debate” but after this they do not put even a single reference to justify such a strong statement. Either the wording is supposed to be changed or at least a reference (such a review paper in the literature) should be put in support of the sentence. Furthermore, this statement contradicts the rationale of the paper: do we have literature on the link or do we have not?

 Always in the theoretical framework section, the second and third paragraphs are devoted to quote literature on the role of innovation. As already observed, the literature is quoted properly, but the connection between innovation-markup-inequality comes somehow without explaining the connection of the argument with the scope of the paper. Again I am not objecting that the issue is relevant for the research: I am interested in seeing how authors want to make this relationship evident in the model they are proposing, and I would like to see it justified better at least in the theoretical part (because in the empirical model the interaction authors put in their model implies a lag structure which is just opposite to the one they suggest in the theoretical section).

 I could also comment other pieces in the theoretical framework section, but I just restrain to the main  point I want to make: the literature is quoted with the correct interpretation; and the one which is quoted belong to the conventional standards, in this respect is then “appropriate”; but the connection of the literature and the thesis authors want to support is weak: which is the thesis? If institutional frameworks make the difference (and I agree), if different sectors have different impact on innovation-markups-inequality (and I agree), if individual motivation affects trust in institutions (and I agree)… why do they totally disappear from the empirical model? If, in contrast, the model is the core of the paper, then what is the point to quote a literature which is not functional to the scope of the paper…      

 Comments 2 and 4 (i.e. study design, and statistical analysis):  the comments are direct consequences of point one. I like, and I agree with the general intuition behind the paper. I think authors correctly highlight the relevant issues, but they need to refine the exposition. In my view they could pursue two ways. Strategy one. Authors may start in general terms, putting the main issues in the present paper, keeping the theoretical framework basically with the concepts it contains now; then before the beginning of the method section they state clearly the variables they want to consider and explain why things which are relevant are supposed to disappear.

 Strategy two. Authors are supposed to cut important (I mean scientifically relevant and intriguing research questions) parts of the theoretical section and restrain themselves only to the specific issues they are going to analyze. But in this case, they are supposed to provide a more refined and specific literature review (I expect the literature quoted in the papers I am going to put at the end of my comments would be helpful in this respect).

 I have 5 more specific observations to draw with respect to the statistical analysis. The first four are “matters of fact”, the fifth is “matter of preference in empirical modelling”

  

1. Please check the coherence between figure 2 and the paragraph at page 10 where the picture is commented, because it seems to me that China has problems (either the comment or the picture is not correct)
2. The last paragraph at page 10 (the one starting “While the relatively stable increase in markups…”) is put in the wrong place, as the section on inequality comes next. Furthermore, the meaning of the sentence is problematic because it contradicts the thesis are supposed to support or indagate. Basically, authors draw a conclusion which is not even supported by the picture (a picture provided would not be enough to prove the statement, i.e. my point is about contradiction and/or self-referentiality of the sentence).
3. At page 11 beginning of the paragraph markup vs Gini index, I hardly can see in the picture what authors see. Again, I ask authors to check the coherence between the words and the lines provided in the pictures.
 
4. Last, but not least. The descriptive exercise shows quite clearly that the three subsets have different behaviors and different magnitude in the period observed. Given the evident heterogeneity in the descriptive analysis, it might be worth considering whether a single model sufficiently captures these differences or if a segmented approach could be more appropriate. It is obvious that insignificant correlations may simply be due to the fact that there is too much heterogeneous variance that compensate away each other…

Fifth observation: What I said with respect to the review of theoretical literature, comes reinforced with respect to the empirical model. I think that authors now miss the opportunity to “reach the point”. The idea of analyzing different institutional frameworks is valuable, as it highlights how innovation, markups, and inequality interact differently across contexts, implying that policy recommendations should not be one-size-fits-all. In contrast, not even an “evergreen dummy” is put in the model to capture all the three-facet heterogeneity authors have chosen and portrayed during the overall length of the paper…

 Other methods may be used for estimation, but in this case I don’t want to make any criticism as any author basically picks up the model which is more comfortable to his/her preferences.

 Comment 5: about conclusions. Because of the observation just made, conclusions are there; they are coherent with the estimated model; but they are not novel. All the set of policy suggestions authors bring about are known in the literature and estimated with previous models… It is exactly because of this that I suggest refining the structure. I think the venue authors have selected is a fruitful path, but I ask them to highlight better the key they already hold. A little precision effort towards a significant gain.****

****

 Minor comments: I would suggest to drop footnote 3 (economic mobility does not need to be defined in an economic paper, and the definition is no precise anyway); I would also suggest to drop footnote 4 for the same reason; I would drop that Ecuador is in Latin America at page 7; I would change the wording because thesis is not hypothesis at page 5 beginning of paragraph 6 (“we hypothesise….”); authors mention “classical economists” but obviously they imply “nowadays mainstream economists” … in this case a footnote to qualify the use of the term would be appreciated.

 Possible references follow to be added and coherent with the points I highlighted as drawbacks now in the paper 

- https://www.proquest.com/openview/89ec0cccad7a3ead8f477b04d0a65312/1?pq-origsite=gscholar&cbl=18750&diss=y
- [Ref 1-7]

Is the work clearly and accurately presented and does it cite the current literature?

Partly

If applicable, is the statistical analysis and its interpretation appropriate?

Partly

Are all the source data underlying the results available to ensure full reproducibility?

Yes

Is the study design appropriate and is the work technically sound?

Partly

Are the conclusions drawn adequately supported by the results?

Partly

Are sufficient details of methods and analysis provided to allow replication by others?

Yes

Reviewer Expertise:

Macroeconomic modelling under limited information and heterogeneity, Financial Instability, Financial sustainability and Inequality, growth cycles (or AD led growth models)

I confirm that I have read this submission and believe that I have an appropriate level of expertise to confirm that it is of an acceptable scientific standard, however I have significant reservations, as outlined above.

## References

[1] : Aggregate Markup and Its Impact on Income Inequality: Country Panel Evidence. *International Economic Journal* .2023;37(3) : 10.1080/10168737.2023.2239204387-400 10.1080/10168737.2023.2239204

[2] : Markups and income inequality: Causal links, 1975-2011. *Journal of Comparative Economics* .2021;49(2) : 10.1016/j.jce.2020.12.002290-312 10.1016/j.jce.2020.12.002

[3] : Rising Corporate Power and Declining Labor Share in the Era of Chicago School Antitrust. *Journal of Economic Issues* .2021;55(2) : 10.1080/00213624.2021.1908802397-407 10.1080/00213624.2021.1908802

[4] : Accounting for the Effect of Government Spending on Income Inequality: Role of Markup Dynamics. *Korea and the World Economy* .2021;22(3) : 10.46665/kwe.2021.12.22.3.129129-157 10.46665/kwe.2021.12.22.3.129

[5] : Competition and Inequality. *Department of Economics Discussion Paper Series. University of Oxford* . https://ora.ox.ac.uk/objects/uuid:b290a4f5-d21d-45a1-96c1-a6c567138f3e 10.2139/ssrn.3652023

[6] : MARKUPS, TAXES, AND RISING INEQUALITY. https://shs.hal.science/halshs-03832267/

[7] : OPTIMAL TAXATION AND MARKET POWER. *SSRN Electronic Journal* .2024; 10.2139/ssrn.4770758 10.2139/ssrn.4770758

# Response

**Published:** 2025-03-25

**Authors:** William Pacheco

**Affiliations:** Economics, University Anahuac Mexico, Huixquilucan de Degollado, State of Mexico, Mexico

Thank you for the detailed and constructive feedback during the peer review process. I would like to confirm that I have carefully addressed all suggestions and comments raised by the reviewer. Specifically, I’ve refined the argumentation, strengthened the discussion by including the recommended references, thoroughly revised data and figures for clarity and consistency, and reformulated the conclusions to highlight the paper’s contributions. I remain available for further suggestions or revisions and look forward to continuing with the editorial process.

 Thank you again for the opportunity to enhance the quality of this manuscript.
